# A Review of Quantitative Structure–Activity Relationship (QSAR) Models to Predict Thyroid Hormone System Disruption by Chemical Substances

**DOI:** 10.3390/toxics13090799

**Published:** 2025-09-19

**Authors:** Marco Evangelista, Ester Papa

**Affiliations:** 1 QSAR Research Unit in Environmental Chemistry and Ecotoxicology, Department of Theoretical and Applied Sciences, University of Insubria, Via J.H. Dunant 3, 21100 Varese, Italy; 2 Department of Science and High Technology, University of Insubria, Via Valleggio 11, 22100 Como, Italy

**Keywords:** endocrine disruption, thyroid hormone system disruption, new approach methodologies, QSAR, MIE, AOP, molecular descriptors, mechanistic interpretation, applicability domain

## Abstract

Thyroid hormone (TH) system disruption by chemicals poses a significant concern due to the key role the TH system plays in essential body functions, including the metabolism, growth, and brain development. Animal-based testing methods are resource-demanding and raise ethical issues. Thus, there is a recognised need for new approach methodologies, such as quantitative structure–activity relationship (QSAR) models, to advance chemical hazard assessments. This review, covering the scientific literature from 2010 to 2024, aimed to map the current landscape of QSAR model development for predicting TH system disruption. The focus was placed on QSARs that address molecular initiating events within the adverse outcome pathway for TH system disruption. A total of thirty papers presenting eighty-six different QSARs were selected based on predefined criteria. A discussion on the endpoints and chemical classes modelled, data sources, modelling approaches, and the molecular descriptors selected, including their mechanistic interpretations, was provided. By serving as a “state-of-the-art” of the field, existing models and gaps were identified and highlighted. This review can be used to inform future research studies aimed at advancing the assessment of TH system disruption by chemicals without relying on animal-based testing, highlighting areas that require additional research.

## 1. Introduction

The endocrine system is a network of glands and organs responsible for the proper production and homeostasis of hormones that control and regulate essential physiological processes, including growth, metabolism, and reproduction [1,2]. While the proper function of this intricate network is vital for maintaining hormonal homeostasis, the endocrine system is vulnerable to exogenous chemical substances known as endocrine disrupting chemicals (EDCs) [1,2]. By mimicking or blocking hormone activity, EDCs cause a wide range of severe adverse health outcomes in living organisms, including, among others, cancers and infertility [1,3,4]. This breadth and severity of effects have made exposure to EDCs a global concern for ecosystems and human health [4,5,6].

In mammals, three major axes characterise the endocrine system: the hypothalamic–pituitary–gonadal (HPG) axis, the hypothalamic–pituitary–adrenal (HPA) axis, and the hypothalamic–pituitary–thyroid (HPT) axis [2,6,7]. The HPT axis regulates the synthesis and release of specific hormones, i.e., thyroid hormones (TH), through a negative feedback loop, ensuring their homeostasis and appropriate physiological concentrations [8,9]. THs, primarily thyroxine (T4) and triiodothyronine (T3), are essential for regulating and coordinating a wide spectrum of physiological processes throughout all life cycle stages, from embryonic development to adult tissue functions. These processes include, among others, the regulation of metabolism and energy balance [10,11], and the influence on the immune, nervous, skeletal, reproductive, and cardiovascular systems [12,13,14,15,16]. Although proper TH activity is essential for normal physiological processes in adulthood [17], its importance is critically pronounced during gestation and early life stages, as THs play a lead role in placenta, brain, and nervous system development [18,19,20,21]. TH system-disrupting chemicals (THSDCs) are a specific subset of EDCs which target the TH system and interfere with the synthesis, secretion, distribution, and metabolism of THs and, ultimately, with their binding to nuclear TH receptors (TRs) for inhibiting or activating gene transcription [22,23]. To date, multiple chemical substances have been recognised as THSDCs, including polychlorinated biphenyls (PCBs), polybrominated diphenyl ethers (PBDEs), perchlorate, bisphenol A, phthalates, dioxins, pesticides, per- and polyfluoroalkyl substances (PFAS), and metals [22,24,25,26]. Exposure to TSHDCs can disrupt TH homeostasis, resulting in cognitive and neurobehavioral disorders [27], cancer [28], and immune, cardiovascular, and reproductive system dysfunctions [29,30,31,32]. Therefore, it is of utmost importance that THSDCs are identified without delays [23,33].

In the framework of the European Green Deal [34] and the Chemicals Strategy for Sustainability [35], the development and implementation of new approach methodologies (NAMs), including in vitro assays and in silico approaches, are heavily promoted to support the identification of EDCs and reduce the reliance on vertebrate animal testing [36,37,38]. The European Union (EU) is advancing this field by funding key dedicated research projects, such as the European Cluster to Improve Identification of Endocrine Disruptors (https://eurion-cluster.eu/). At the international level, the Organisation for Economic Co-operation and Development (OECD) included in vitro and in silico methodologies in the “Conceptual Framework for Testing and Assessment of Endocrine Disrupting Chemicals” as a relevant source of information to assess the ED properties of substances [39].

In previous years, the criteria for the determination of ED properties has been adopted under the main EU chemical regulations, such as Regulation (EU) No 528/2012 [40], Regulation (EC) No 1107/2009 [41], and Regulation (EC) No 1272/2008 [42]. Although there are minor differences in the terminology across these regulations, a chemical substance is recognised as an EDC if it meets the following three criteria: (i) it shows an adverse effect, (ii) it can alter the endocrine system through an endocrine mode of action, (iii) a plausible link between (i) and (ii) must be established. In this regard, the combined application of NAMs and the adverse outcome pathway (AOP) framework [43] has been suggested as an effective strategy [36,37,44]. Firstly, the development and application of NAMs can identify molecular initiating events (MIEs) in AOPs through which chemical substances can trigger specific endocrine modes of action and consequently lead to endocrine-related adverse effects. Secondly, a biologically plausible link between endocrine modes of action and adverse effects can emerge. This synergy gains even greater significance as it is now established that EDCs can disrupt various pathways involving hormone signalling, rather than the initial belief that their effects were solely mediated by interacting with nuclear receptors [7]. As is the case with other types of NAMs, a synergism between AOPs and quantitative structure–activity relationship (QSAR) models has been established [23,45,46]. The AOP network for TH system disruption developed by Noyes et al. [47] holds significant importance in the field, as it was used as foundational framework by the European Union Reference Laboratory for alternatives to animal testing (EURL ECVAM) to validate a suite of mechanistic in vitro assays for identifying THSDCs [33,48]. Multiple MIEs have been well documented, which involved each step of the TH cycle [47]. Examples include, among others, the inhibition of thyroperoxidase (TPO), which is a critical enzyme for TH synthesis as it catalyses tyrosine residue iodination; binding to serum TH distributor proteins, such as transthyretin (TTR), thyroid binding globulin (TBG), and albumin, which serve as buffers of TH in the bloodstream to ensure the proper TH concentration in their free form; and binding to TRs, which are proteins that, once bound to TH, regulate gene expression and ultimately biological effects [47].

Despite the growing need and interest to advance TH system disruption assessments using in silico and QSAR approaches, a comprehensive review on this topic is currently lacking. While valuable studies have been recently published [49,50], their scopes were different. Sellami and co-workers presented a review on in silico studies focused on nuclear receptors, covering a range of approaches that included not only QSARs but also other methods such as molecular docking and dynamics, and considered the TR as the sole target related to the TH system [49]. In contrast, Vergauwen and co-workers presented a broader review focused on in vivo, in vitro, and in silico methods currently available for TH system disruption assessment [50]. However, their specific examination of in silico tools was confined to models available in open-source predictive tools (e.g., Danish (Q)SAR Database), leading to the identification of twelve models [50]. The present review addressed the current state-of-the-art of QSAR models published in the literature from 2010 and up to 2024 to predict potential TH system disruption by chemical substances. This allowed for a detailed characterisation of how this field has evolved over time, which type of TH system-related endpoints were modelled (and not) by these models, the main data sources used for model development, the modelling approaches, the applicability domain (AD) definitions, which types of chemicals have been assessed, which types of molecular descriptors have been selected as more relevant, and their mechanistic interpretations to suggest potential biological mechanisms. Mapping out the state-of-the-art on this topic is necessary to consolidate existing knowledge, identify research gaps, and offer a resource to guide future investigations. To provide the most up-to-date perspective on the topic, a separate paragraph is dedicated to key articles published between January and July 2025. The decision to treat these publications separately was made because 2025 is an incomplete year and a full comprehensive review of its literature would be premature.

## 2. Materials and Methods

### Criteria of Inclusion and Exclusion and Literature Collection

To meet the scope of this review, the following specific inclusion and exclusion criteria were predefined to collect relevant publications. (1) Original peer-reviewed research articles published from 2010 and up to 2024, where new QSAR models for predicting the potential TH system disruption by chemical substances were proposed. Original peer-reviewed research articles not proposing a new QSAR model (e.g., experimental studies, the application of unsupervised learning methods) were excluded. (2) Modelling efforts focused on predicting MIEs within AOPs for TH system disruption; the AOP network proposed by Noyes et al. was used as a reference [47]. MIEs, such as the induction of the constitutive androstane receptor (CAR), pregnane X receptor (PXR), aryl hydrocarbon receptor (AhR), and peroxisome proliferator-activated receptor (PPAR), were not considered in this review as they were not identified as being thyroid-specific by Dracheva et al. [51] in a study following that by Noyes et al. [47], and were also not addressed by the EURL ECVAM [33,48]. (3) From articles reporting multiple models for the same endpoint, only the QSARs explicitly identified as the best ones by the developers and/or applied for screening purposes within the same study were retained, thereby excluding QSARs arising from, e.g., different data partitioning, data imbalance handling techniques, and feature selection procedures (please note that a description about the effects of such approaches on models’ performances was provided Section 3.5). (4) Original, peer-reviewed research articles focusing on QSAR development for a series of “selective ligands” in illness treatments or drug development were excluded. The same inclusion and exclusion criteria were applied to identify relevant articles published between January and July 2025.

The literature search was conducted using the Web of Science database, according to the inclusion and exclusion criteria. To obtain a more comprehensive collection of relevant publications, the literature search was conducted using both the full names and abbreviations of key biological targets (e.g., TTR, TPO) as keywords, rather than searching for each specific MIE (e.g., TTR binding, TPO inhibition) [47,48,51]. Hereafter in this review, each biological target will be referred to as the related MIE. The search strategy involved the following combinations of keywords: “thyroid system” AND “QSAR”, “thyrotropin releasing hormone receptor” AND “QSAR”, “TRHR” AND “QSAR”, “thyroid stimulating hormone receptor” AND “QSAR”, “TSHR” AND “QSAR”, “thyroperoxidase” AND “QSAR”, “TPO” AND “QSAR”, “sodium iodide symporter” AND “QSAR”, “NIS” AND “QSAR”, “type 1 deiodinase” AND “QSAR”, “DIO1” AND “QSAR”, “type 2 deiodinase” AND “QSAR”, “DIO2” AND “QSAR”, “type 3 deiodinase” AND “QSAR”, “DIO3” AND “QSAR”, “deiodinase” AND “QSAR”, “DIO” AND “QSAR”, “iodothyronine deiodinase” AND “QSAR”, “IYD” AND “QSAR”, “iodotyrosine deiodinase” AND “QSAR”, “DUOX” AND “QSAR”, “dual oxidase” AND “QSAR”, “pendrin” AND “QSAR”, “monocarboxylate transporter 8” AND “QSAR”, “MCT8” AND “QSAR”, “monocarboxylate transporter 10” AND “QSAR”, “MCT10” AND “QSAR”, “monocarboxylate transporter” AND “QSAR”, “MCT” AND “QSAR”, “organic anion transporter polypeptide 1C1” AND “QSAR”, “OATP1C1” AND “QSAR”, “organic anion transporter polypeptide 1A4” AND “QSAR”, “OATP1A4” AND “QSAR”, “organic anion transporter polypeptide” AND “QSAR”, “OATP” AND “QSAR”, “multidrug resistance protein 1” AND “QSAR”, “MDR1” AND “QSAR”, “multidrug resistance associated protein 2” AND “QSAR”, “MRP2” AND “QSAR”, “thyroid binding globulin” AND “QSAR”, “TBG” AND “QSAR”, “transthyretin” AND “QSAR”, “TTR” AND “QSAR”, “albumin” AND “QSAR”, “thyroid receptor” AND “QSAR”, “TR” AND “QSAR”.

The selection of relevant publications from this search followed two main phases. An initial screening of titles and abstracts was conducted to assess relevance based on the inclusion and exclusion criteria. If relevance could not be determined from this step, a full-text analysis was performed.

## 3. Results and Discussion

The final list comprised thirty publications including eighty-six distinct QSAR models. A summary is reported in Table 1, where studies are presented chronologically. Additional information is reported in Table S1 in the Supplementary Materials.

### 3.1. Temporal Trend

**Figure 1:**
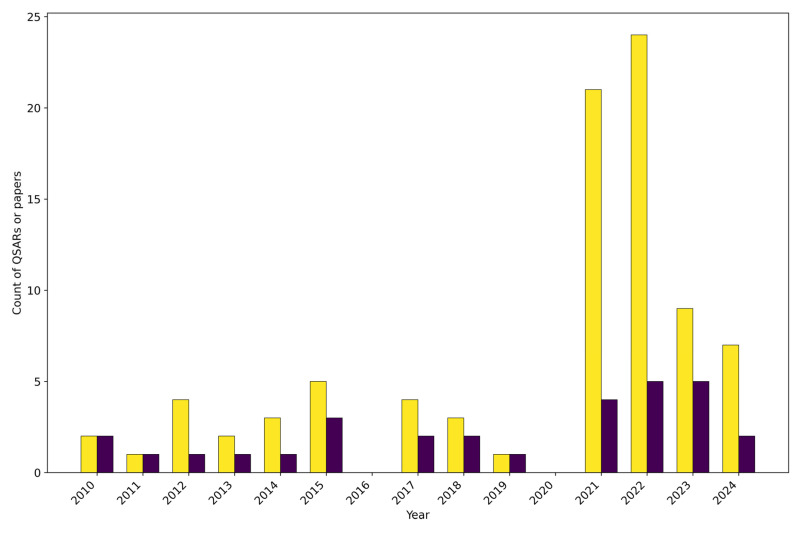
Annual distribution of QSAR models (yellow bars) and papers (purple bars).

Figure 1 illustrates the number and distribution of the selected QSAR models and papers over time. Despite minor fluctuations, modelling efforts remained relatively stable since 2010 until 2020, followed by a noticeable surge in the period 2021–2022 and by a slight decrease in 2023–2024. Sixteen out of the thirty papers selected in this review were published in the period 2021–2024, suggesting a recent acceleration of research into TH system disruption using QSAR-based approaches. Before this, the field was characterised by notably sparser publications, with fourteen papers appearing over the ten years from 2010 to 2020. Notably, no relevant publications were detected in 2016 and 2020, which could signify periods of reduced research focus (e.g., the impact of the COVID-19 pandemic), or a shift in research priorities. The number of developed QSAR models mirrors this trend. Indeed, while a year-to-year fluctuation was observed up to 2020, over 70% of the total QSARs were published within the last four years, with a pronounced surge occurring in 2021 and 2022. The number of QSAR models exceeding the number of publications is largely attributed to the increasing practice of proposing multiple models within a single publication, often addressing, for instance, different endpoints, descriptor types, and/or methodological approaches. These findings could be mainly attributed to the growing availability of publicly available high-throughput screening (HTS) data for multiple thyroid-related endpoints, such as those from large-scale projects like Toxicity Forecaster (ToxCast) (https://www.epa.gov/comptox-tools/toxicity-forecasting-toxcast) and Toxicology in the 21st Century (Tox21) (https://tox21.gov/).

### 3.2. Modelled MIEs

The selected QSAR models were developed for eleven different MIEs for TH system disruption, which represent only a subset of the over twenty described by Noyes et al. [47]. MIEs regarding DUOX, IYD, and pendrin inhibition, as well as those related to cellular TH transport (i.e., MCT8, MCT10, OATP1C1, OATP1A4, MDR1, and MRP2), have never been addressed by QSAR modelling.

**Figure 2:**
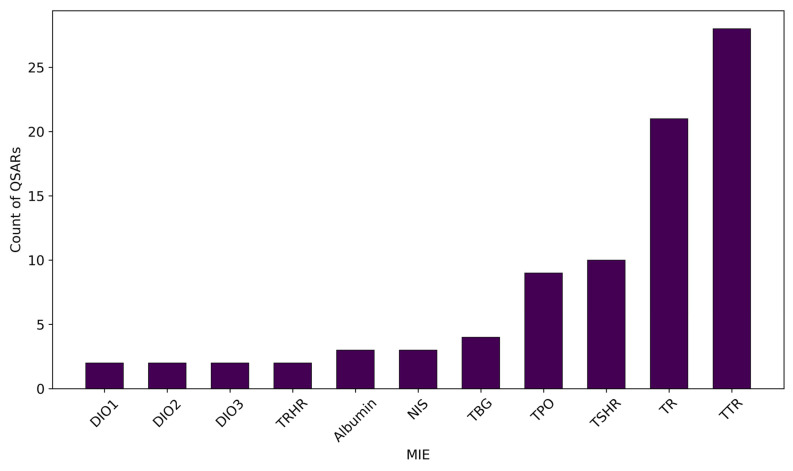
Count of QSAR models developed for each MIE.

As illustrated in Figure 2, a predominant focus was placed on TR and TTR, which together account for 57% of all the QSARs included in this review. This large number could be attributed to the widespread availability of in vitro data for these MIEs and to their established mechanistic links with TH system disruption [7]. While less frequently modelled than TTR and TR, targets like TSHR and TPO were still relatively well represented. The modelling of TPO, which is a key enzyme for THs synthesis, and TSHR, which is a protein that regulates thyroid gland function, highlighted an expanding scope of investigation beyond just TH distribution or nuclear receptor binding reflected by, respectively, TTR and TR. In contrast, other important MIEs remained significantly poorly addressed, highlighting the notable gaps in the current research in the field. The critical roles of albumin, TBG, NIS, TRHR, and the three deiodinases (DIO 1, 2, and 3) in TH synthesis, distribution, and metabolism are well established [31,47]. However, despite their recognised relevance, the scarcity of QSAR research for these targets pointed out potential challenges, such as poor data availability or a limited interest or awareness among QSAR developers. This almost-negligible modelling effort for these MIEs indicates a significant opportunity for future research and QSAR model development.

**Figure 3:**
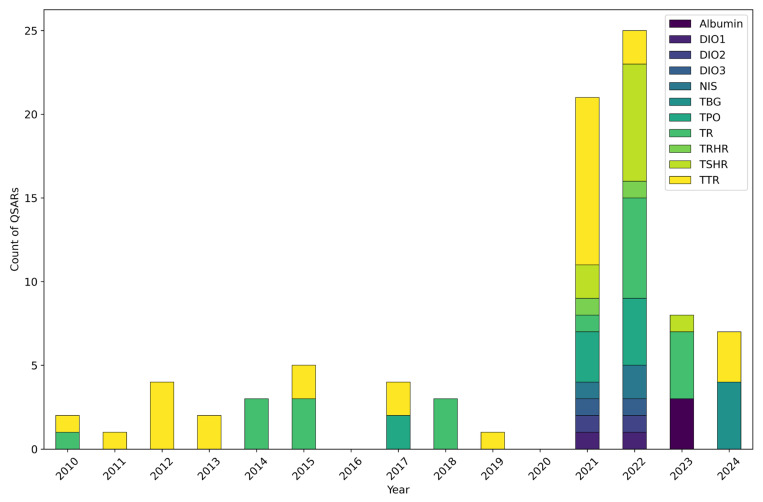
Annual distribution of QSAR models, categorised by MIE.

As illustrated in Figure 3, TR and TTR were consistently modelled throughout the entire study period, reflecting their long-standing recognition as key targets for TH system disruption assessment. A shift in research focus is evident from 2021 onward, with a significant diversification of modelled MIEs. Specifically, the modelling efforts on TSHR, TPO, NIS, TRHR, and deiodinases (DIO1, DIO2, DIO3), though less numerous overall, were distinctly concentrated in 2021 and 2022. This concentrated activity, however, largely stemmed from two studies by Dracheva et al. [51] and de Lomana et al. [119], where multiple endpoints were addressed in the same publication. QSARs addressing other important TH distributor proteins, i.e., TBG and albumin, were only published in the last two years.

As discussed in Section 3.1, this trend of diversification and the surge in the 2021–2022 biennium are likely linked to the growing availability and accessibility of HTS data. Prior to 2021, the scarcity of QSAR studies for MIEs other than TR and TTR likely stemmed from a combination of factors: a scarcity of available experimental data (for instance, Gadaleta et al. [104] pointed out that MIEs such as MCT8, MCT10, and OATP1C1 lacked sufficient active compounds in the ChEMBL database to be used for modelling purposes) and a complexity of developing suitable and validated assays for their generation, or a lower awareness of the mechanistic role of these MIEs in TH system disruption. It is worth highlighting that no in vitro assays for TH system disruption have yet been validated by the OECD [33,48,149,150], which might slowing data generation. The relatively recent publication of the AOP network for TH system disruption by Noyes and colleagues [47] likely played a crucial role. By providing a more structured understanding of these diverse pathways, it stimulated research into previously underexplored MIEs. The growing number of publications and QSARs covering multiple MIEs underscored the increasing awareness of the multifaceted and interconnected nature of TH system disruption.

### 3.3. Data Sources

**Figure 4:**
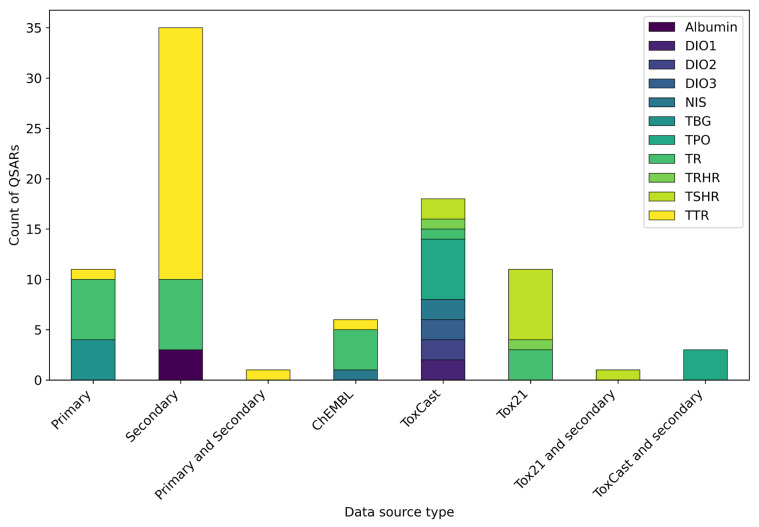
Count of QSAR models based on data source type, categorised by MIE.

As detailed in Table 1, the QSARs selected for this review were based on data from three main source types: (1) primary sources, where data was generated as part of the same study; (2) secondary sources, where data was collected from the existing literature; (3) publicly available databases (i.e., ToxCast, Tox21, and ChEMBL). In most cases, these sources were used individually, while in others, they were combined (Figure 4).

The data source reference(s) used to develop each QSAR are reported in Table 1. The data included in publicly available databases served as unique data sources for developing thirty-five distinct QSARs, representing approximately 41% of the total. MIEs covered by these QSARs included TTR, TR, TSHR, TPO, TRHR, NIS, and the three deiodinases. With a single exception [121], all of the studies using data from the ToxCast and Tox21 projects were published from 2021 to 2023, proposing all of the available QSARs addressing TPO, NIS, TSHR, TRHR, DIO1, DIO2, and DIO3. As previously discussed, these findings were linked to the growing availability and accessibility of comprehensive HTS datasets. This data availability, combined with an increasing awareness of the critical roles these targets play within the TH system, has broadened the scope of QSAR investigations for TH system disruption assessment.

In contrast, primary and secondary data sources were used alone for the development of forty-six (53%) QSARs. The consistent use of primary and secondary data sources from the literature throughout the entire study period underscored their sustained importance. These models covered a stricter range of MIEs, such as TR and TH distributor proteins TTR, TBG, and albumin, underscoring limited data availability or utilisation for other MIEs. Whilst the majority of these QSARs were developed using data from in vitro experiments, Kowalska et al. [90] and Yang et al. [52] developed a total of six QSARs to predict binding energies to TTR and TBG, respectively. Binding energies used for models’ development were generated within the same studies through molecular docking and dynamic simulations and used as dependent variables. The successful application of integrated in silico approaches highlighted their utility as an effective strategy when experimental data from in vivo or in vitro studies is limited or entirely lacking, further enabling the exploration of complex molecular interactions that might be otherwise inaccessible.

A key aspect across the studies was data transparency. The data sources and data used for model development were consistently made available, either directly within the publications or through adequately referenced sources. This commitment to data availability aligned with the FAIR (Findable, Accessible, Interoperable, Reusable) principles for data sharing [151], thereby optimising data reuse for future research.

### 3.4. Chemical Classes

The datasets used for QSARs training and validation included either structurally heterogenous chemicals or class-specific chemicals.

**Figure 5:**
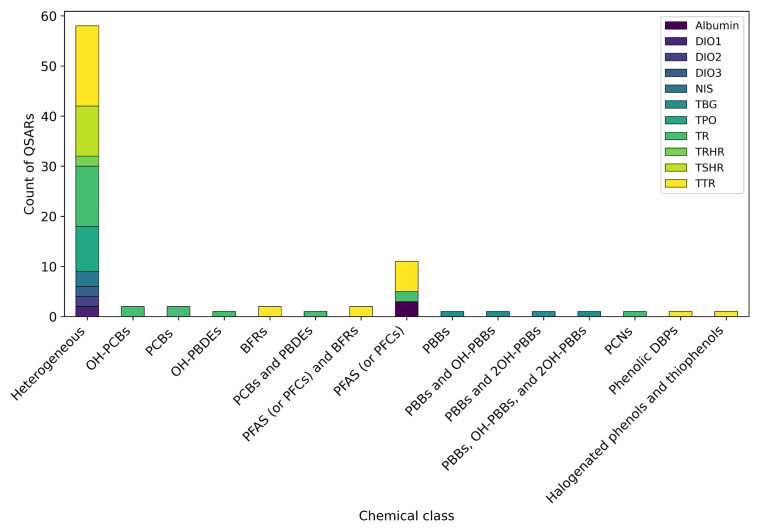
Count of QSAR models based on structurally heterogenous or class-specific datasets, categorised by MIE.

Structurally heterogeneous datasets were used for approximately 67% of the QSARs. These datasets primarily consisted of organic chemicals, encompassing a mix of environmental pollutants, natural compounds, and, occasionally, drugs. The sizes of such datasets varied considerably, from 41 to 8682 compounds. About 83% of these QSARs were published within the last four years, reflecting the spreading availability of HTS data, as previously described. As illustrated in Figure 5, all the endpoints were addressed using heterogeneous datasets, with the exception of TBG and albumin.

In contrast, only a limited number of chemical classes have been tested and modelled for TH system disruption, addressing a limited number of MIEs. These datasets primarily focused on environmental pollutants of known concern, including PCBs and their hydroxylated metabolites, PBDEs and their hydroxylated metabolites, PCNs, halogenated phenols and thiophenols, phenolic DBPs, PFAS (often referred to as PFCs), and PBBs and their hydroxylated metabolites. The sizes of these datasets were generally smaller compared with the structurally heterogeneous ones, ranging from 17 to 107 compounds. Furthermore, these data were exclusively generated within the same study or retrieved from the existing literature, hence were never extracted from databases. Notably, only TR and TH distributor proteins (i.e., TTR, TBG, and albumin) were modelled using these datasets, underscoring limited data availability or the utilisation of specific class data for other thyroid-related endpoints. It is also important to highlight that almost half of these QSARs were published within the last four years. This trend suggested that, despite the increasing availability of HTS data, the reliance on data published in the literature by independent research groups remained critically important.

Although certain compounds, like bisphenol derivatives, phthalates, various pesticides, and constituents of personal care products have been experimentally identified as THSDCs [22,25,26,152], many others within these same classes remain poorly addressed. This lack of data is concerning because structural similarity among compounds within the same class may suggest a similar toxic potential. This highlighted a strong need for additional in silico or in vitro efforts to generate more data for these and other chemical categories for specific MIEs. Broadening the chemical space coverage for each of these chemical categories would be essential to develop new, specific QSAR models, enabling a more robust hazard assessment for entire groups of compounds. Building on the successful application of integrated in silico approaches by Kowalska et al. [90] and Yang et al. [52], as described in Section 3.3, a similar approach could be an effective strategy to address other MIEs for specific chemical classes.

Generally, the use of heterogeneous datasets can improve a model’s AD coverage and generalizability for large screening applications. In contrast, local QSARs, which are specifically designed for specific classes of compounds, are often preferred for their ability to more accurately capture subtle structural differences and specific structure–activity relationships. This can lead to (potentially) more reliable predictions within that defined chemical space. Therefore, the choice between using global or local QSAR models depends on the specific application purposes. Furthermore, the inherent complexity of heterogeneous data can hinder the mechanistic interpretation of molecular descriptors (see Section 3.8). When a model is trained on a wide array of chemical structures, it is more challenging to pinpoint the exact structural features or physicochemical properties responsible for a particular activity. This is in contrast to datasets of specific classes, where a clearer structure–activity relationship can emerge, making interpretation more straightforward.

### 3.5. Modelling Approaches

A wide variety of modelling algorithms are available for QSAR model development. These range from traditional methodologies, such as MLR and LDA, to more complex machine learning methodologies, such as NN and SVM [46,153]. The choice of algorithm generally depends on the complexity of the data and the desired interpretability of the model. Thus, the landscape of algorithms for QSAR development lacks a universally accepted solution, as each method presents its own set of strengths and limitations.

**Figure 6:**
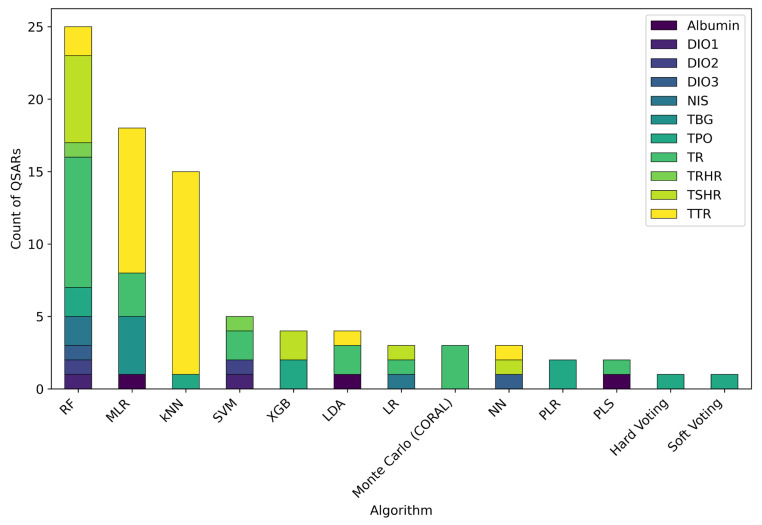
Count of QSAR models based on the modelling algorithm, categorised by MIE.

As illustrated in Figure 6, different modelling algorithms and approaches were identified across the papers.

Over two-thirds of the QSARs selected in this review (67%) were designed for classification, a preference largely driven by the nature of HTS data. Large-scale projects like ToxCast and Tox21 generate vast datasets, where the effect on a biological target by compounds is often reported with a simple categorical outcome, i.e., “active” or “inactive”. This format has consequently led to a shift in QSAR modelling for TH system disruption, favouring classification-based approaches over regression-based ones.

RF was the most frequently used algorithm, followed by MLR and kNN. Overall, RF, kNN, and MLR were used to develop a total of fifty-eight different QSARs, corresponding to approximately 67% of the total models. It is important to highlight that a single study by Dracheva et al. [51] utilised RF to develop eleven different QSARs for the prediction of nine MIEs, which significantly influenced the overall count of RF applications.

The majority of studies concentrated on a single, well-defined modelling strategy, while a few explored more comprehensive approaches, systematically exploring combinations of algorithms, descriptor types, or class-balancing techniques to achieve the best possible performance. While a comprehensive comparative analysis of the predictive models’ performances would be highly valuable, it fell outside the scope of this review, as it was hindered by the following two key reasons. Firstly, the distribution of available models was highly imbalanced. While MIEs like TTR and TR have been extensively studied with multiple QSARs, others have been addressed by a few, or even no, models. Secondly, cross-study comparisons of models’ performances can be performed only when the same dataset and data processing technique are used [154], meaning that simply looking at the statistical metrics of QSARs from different papers would be inappropriate to determine which modelling approach is truly superior. For example, Schür et al. recently reviewed predictive ecotoxicology studies and concluded that no existing studies were truly comparable due to inconsistent methodologies regarding datasets, data processing, and performance statistical metrics [155]. This finding could also be applicable to the broader toxicological context. Therefore, the focus of this section was placed on studies that directly explored various modelling approaches, in terms of algorithms, descriptor types, or data-balancing methods, on a single, consistent dataset. This approach allowed the authors to conduct a reliable assessment of which specific methodology yielded the best predictive results.

All of the models for TPO inhibition were developed using structurally heterogenous datasets of chemicals (see Table 1 and Figure 5). Rosenberg et al. [121] developed two robust QSAR models using PLR, named QSAR1 and QSAR2, using an initial selection of predefined molecular descriptors and training set-dependent scaffolds. The authors evaluated seven different modelling strategies, including approaches that used scaffolds and those that did not, in both single and composite models. The most successful strategy was a composite model that uniquely combined a single, unbalanced model with balanced sub-models from a composite one. This strategy was found to be particularly effective in handling the challenges posed by imbalanced datasets, and led to the final QSAR1 and QSAR2 models (with a cross-validation balanced accuracy equal to 80.6% and 82.7%, respectively). Similarly, Seo et al. [107] developed binary, ternary, and quaternary QSAR models. They applied multiple algorithms, such as RF, SVM, artificial NN, Adaptive Boosting (AdaB) and XGB, and hard- and soft-voting classifiers. Each algorithm was combined with multiple categories of fingerprints (FPs) (e.g., Morgan FPs, Atom Pair Count FPs) and dimensionality reduction techniques (i.e., principal component analysis (PCA) and LDA) to address overfitting. The Atom Pair Count FPs was the best-performing FP, whereas the best-performing models in the binary, ternary, and quaternary models were the hard-voting classifier, XGB with LDA, and soft-voting classifier, respectively (test scores equal to 0.66, 0.51, and 0.52, respectively). Gadaleta et al. [120] applied multiple algorithms, including SVM, balanced RF, RF, and kNN, and explored different partitioning schemes to stratify and select active compounds in different ways. The top-performing models were based on balanced RF and kNN using a dataset that excluded compounds with an ambiguous active categorization. The models achieved a balanced accuracy of 76–78% on external data, which resulted as a performance comparable to the reported experimental variability of the assay used to generate modelled data.

Regarding TR binding, Bai et al. [122] developed classification QSARs based on twenty-two PCBs using LDA and SVM. Both showed strong and equal accuracy in the training set (88.2%), with the SVM model exhibiting a greater accuracy in the test set, equal to 80%. Akinola et al. [91] developed classification models applying LR and LDA on a dataset of sixty-eight OH-PCBs, showing that both methods performed identically (accuracies in the training set and in the test set equal to 84.3% and 76.5%, respectively). Yan et al. [123] developed ternary classification models applying LDA, classification and regression trees (CART), and SVM on a dataset of structurally heterogenous compounds. SVM proved to be the optimal algorithm, with a total accuracy in the training and test set equal to 81.4% and 76.5%, respectively. Sapounidou et al. [113] proposed a comprehensive set of twenty-three QSAR models for various MIEs related to endocrine disruption, including TRβ binding, utilising the conformal prediction (CP) framework combined with RF as the modelling algorithm. Five different data-balancing techniques were employed (for more details, see below), with CP providing the best one. A balanced accuracy equal to 0.78 was achieved.

As for TPO, all of the models for TSHR inhibition were developed using datasets of structurally heterogenous chemicals. Xu and colleagues [106] developed binary classification models comparing three different algorithms: RF, XGB, and LR. Both RF and XGB models showed good predictive performances, with balanced accuracies of 0.85 and 0.84, respectively. The authors further developed a simplified RF model using the seven most influential descriptors, which maintained strong performance (balanced accuracy equal to 0.83). Additionally, they first developed a regression model using MLR, which yielded an R^2^ of 0.35. Therefore, a regression model using XGB was developed and the R^2^ increased up to 0.65. Later, Liu et al. [95] explored various combinations of seven molecular representations (including different types of FPs and Mordred descriptors) and four algorithms (RF, SVM, multilayer perceptron, and graph attention network). The best-performing model was a RF using PubChem FPs, which achieved a balanced accuracy of 0.94 on the validation set.

Regarding TTR binding, Zhang et al. [87] developed QSAR classification models applying kNN, PLS discriminant analysis (PLS-DA), and SVM. The kNN model, with a k-value of 4, showed the best performance, achieving the highest correct classification rate during both internal and external validation (0.88 and 0.82, respectively). Similarly, Rybacka et al. [137] tested seven different machine learning methods (MLR, PLS, associative NN, kNN, RF, SVM, and fast stepwise (stagewise) multivariate linear regression) and five distinct descriptor sets. The best result was obtained by combining the associative NN algorithm with Dragon descriptors, which achieved a balanced accuracy equal to 89%.

Finally, de Lomana et al. [119] used five different algorithms (i.e., LR, RF, XGB, SVM, and NN) in combination with three class-balancing techniques (see below for more details) to predict multiple MIEs. All algorithms performed similarly, with a tendency for the models trained on over-sampled data to achieve better results. Balanced accuracies ranged from 0.68 to 0.82 for different endpoints.

Although the use of diverse algorithms was evident (Figure 6), no clear temporal trend was observed in the type of modelling algorithms employed. This suggested a consistent application of both established and newer algorithms across different publication years, rather than a gradual shift toward more complex techniques. Interestingly, despite the recent advancements in machine learning and deep learning approaches, classical algorithms such as MLR, LDA, and PLS continued to be widely used, given their interpretability and simplicity. Indeed, their added value stems from providing easily understandable models that offer direct insights into the structural features driving the activity, which is a key aspect to enhance confidence with QSARs. On the contrary, the “black box” nature of more complex algorithms makes them less transparent and, if not adequately controlled, potentially more susceptible to overfitting [156]. Therefore, an important research direction is to leverage the power of complex algorithms by focusing on developing methods that enhance their interpretability and transparency, thereby increasing user confidence and facilitating their broader adoption.

Establishing a clear link between the algorithm type and a specific endpoint proved challenging, as most endpoints have been assessed by a few, or even no, QSARs. Regarding TTR and TR, the two most modelled endpoints, algorithms capable of handling linear relationships (e.g., MLR) and non-linear relationships (e.g., kNN) between independent and dependent variables were both utilised, with a slight preference for the second group.

An additional methodological aspect observed across the studies was the application of class-balancing strategies. This is crucial to address class imbalance, where one class (e.g., inactive compounds) is much more common than another (e.g., active compounds) in a training dataset. This imbalance is frequently found in data from databases or generated through HTS and can cause a model to become biased toward the majority class, leading to a poor performance with the minority class. This is especially critical in hazard prediction, where mistakenly predicting a dangerous compound as safe is a far more serious error than the opposite. Several effective strategies exist and were observed in the reviewed studies. Sapounidou et al. [113] in combination with RF, explored five different data-balancing techniques: CP, equal size sampling (under-sampling), over-sampling by duplication, synthetic minority over-sampling technique, and random over-sampling examples. As described above, the use of CP was the best choice. de Lomana et al. [119] combined five different algorithms (i.e., LR, RF, XGB, SVM, and NN) and three class-balancing techniques: weight balancing, over-sampling, and under-sampling. The models trained on over-sampled data achieved better results. Xu et al. [106] employed the synthetic minority over-sampling technique-edited nearest neighbours (SMOTEENN) technique, which combines over-sampling the minority class samples with under-sampling the majority class samples to achieve a more balanced distribution. Gadaleta et al. [104,120] developed models using balanced RF, which is an adaptation of the more traditional RF that incorporates the internal balancing of categories. Finally, Liu et al. [95] employed a threshold moving method. The increasing volume of HTS data highlights the critical importance of effective class-balancing strategies for enhancing the robustness and reliability of models built on these datasets.

### 3.6. Validation Strategies

Validation stands as a crucial step in QSAR model development, ensuring the appropriateness of goodness-of-fit, overall robustness, and predictive ability, thereby ultimately maximising the model’s reliability [157,158,159]. Validation procedures can be distinguished as internal and external. Internal validation is conducted to evaluate the robustness and the predictive ability of a QSAR on the data from which it was developed (i.e., training set). External validation, on the other hand, is conducted to evaluate the actual predictive ability of a QSAR on data not used for its development (i.e., test set). Thus, external validation is of key importance as it assesses the model’s true predictive power using unseen data [157,158,160]. Although the best strategy to perform external validation involves the use of completely new and independent datasets, obtaining these datasets is often challenging given the scarcity of available experimental data. Therefore, a common practice is to partition the available data into a training set and into a test set (i.e., dataset splitting) [157,158,161].

All of the QSARs reviewed in this study underwent some form of internal and/or external validation. Internal validation was performed to evaluate the robustness of seventy-two QSARs, accounting for approximately 84% of the total. The most frequent internal validation strategy was the *k*-fold cross-validation (CV), which was used in fifty-five instances. This approach involves splitting the training set into *k* equally sized groups. The method iteratively trains a model on *k* − 1 groups and validates it on the remaining group. This is repeated *k* times, such that each group serves as the validation set once [162]. In this review, *k* values were typically set to 2, 5, or 10. In some instances, this strategy was often referred to as leave-more-out CV (LMO CV) and as leave-one-out CV (LOO CV). The latter is the simplest case of *k*-fold CV, where each compound of the training set is removed one at time, and it was employed in twenty instances. Finally, the stratified bagging method was used in one instance [137], where *k*-fold CV was also tested. Additionally, regression-based QSARs often underwent further internal validation strategies, such as the QUIK rule [163] to detect high predictor collinearity, Y-randomisation to detect chance correlations [164], and the use of the bootstrapping coefficient [165]. Details about each model are reported in Table S1. External validation was performed to evaluate the predictive ability of eighty-three QSARs, representing almost all of them. It is important to highlight that for the three QSARs where external validation was not conducted, this omission was not an oversight, instead it was intentionally not performed and adequately justified by the authors [93,121]. For example, Rosenberg et al. [121] proposed two QSARs for TPO inhibition, named QSAR1 and QSAR2, which were developed using two independent datasets. QSAR1 was developed using one dataset as the training set and the other one as the test set for external validation. Instead, QSAR2 was developed by merging both datasets to form a larger training set: whilst QSAR2 was developed using the same modelling method and CV approaches as QSAR1, it purposely lacked external validation. Both QSARs showed good performances and were applied to broader screening purposes. In the study by Gallagher et al. [93], given the small size of the dataset (twenty-two compounds), the “Small Dataset Modeler” tool proposed by Ambure et al. [166] was utilised to facilitate an exhaustive double CV approach that uses the entire dataset without requiring splitting it into a training set and a test set, making it an effective and suitable solution to validate QSARs based on limited data. Beyond these three specific exceptions, data partitioning into a training and test set was performed by employing various splitting strategies. Random splitting is a frequently adopted strategy [167], and its prevalence was also observed in this review, where it was applied in sixty-three instances. While straightforward, this procedure can lead to an uneven data distribution particularly when dealing with small-sized datasets or with skewed class distributions [168,169,170]. This imbalance might ultimately result in training and test sets that deviate from the representativeness suggested by Golbraikh et al. [171], who argued that using rationally selected training and test set can enhance QSAR reliability [171]. Alternative partitioning strategies have been proposed and used for a strategic selection of training and test set compounds [157,168]. In this review, strategies alternative to random splitting included those based on sorted response variables [53,90,133,144] or on the Kennard–Stone algorithm [87,91,172]. The details about each model are reported in Table S1.

The diverse array of splitting strategies reflected the fact that there is not a single and widely considered ideal partitioning scheme. Instead, the choice depends on the specific dataset type, its size, and the modelling methodology employed in the study [168]. Encouragingly, with only a few noted exceptions, the predominant practice across the reviewed studies involved the combined application of both internal and external validation. This robust approach, which was utilised in 80% of the QSAR models, suggested a strong commitment within the field to ensure their validity and reliability.

### 3.7. Applicability Domains

A single QSAR model cannot accurately predict the entire chemical universe [173]. Thus, each QSAR needs to be associated with a clearly defined AD. This domain determines whether a QSAR model can provide reliable or unreliable predictions (i.e., extrapolations) based on the structural, physicochemical, and response information present in the training set of the model [157,158,174]. No single, universally accepted method exists for defining the AD of a QSAR model. Instead, a range of methodologies are utilised, each offering a distinct approach [175,176]. These methods can differ in their restrictiveness and can yield either categorical outcomes (e.g., a simple “in” or “out” of the AD) or continuous values (e.g., distance) quantifying the relative position of a compound to the AD boundaries or centre [157].

An alarming finding was that the AD was not explicitly defined for thirty-two QSARs, accounting for approximately 37% of the total models. Among these, it is worth highlighting the studies by Bai et al. [122] and by Akinola et al. [91]. Bai et al. [122] developed two QSARs using a training set of twenty-two PCBs, which were then applied to predict TR binding for the remaining PCBs congeners. Similarly, Akinola et al. [91] developed two QSARs based on TR binding data for sixty-eight mono-hydroxylated PCBs. While the ADs of these models were not formally defined, it is reasonable to assume that they were implicitly limited to these specific chemical classes due to their relatively small number and well-defined congeners. In the publication by de Lomana et al. [119], nine different QSARs were developed without defining a priori their ADs. Instead, ADs were assessed post hoc in terms of the Tanimoto coefficient by comparing the chemical space covered by the training sets with the chemical spaces covered by well-known datasets of pesticides, cosmetics, and drugs.

**Figure 7:**
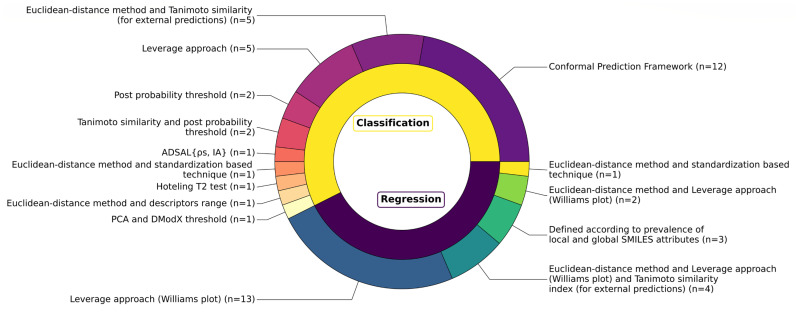
Types of AD definitions used in the selected QSAR models (and count).

Despite its critical importance for ensuring the reliability of predictions for new chemicals, the AD was explicitly defined for fifty-four QSARs (see Table S1). As illustrated in Figure 7, a variety of methodologies were used for the AD definition of both classification and regression QSARs, showing that some studies integrated multiple approaches while others relied on a single method. While some methods were used more frequently, others appeared in only a single instance.

The leverage approach was the most frequently used method, employed in approximately 44% of the QSAR models. This method was used either as a standalone technique [52,53,90,112,144,145,147,148] or in combination with other approaches [57,59,114], often complemented by the Williams plot as a graphical support for AD visualisation [52,53,57,59,90,112,114,144,147,148]. For example, in two different studies by Yang et al. [57,59] the leverage approach was combined with the Euclidean distance-based method to define the AD boundaries of two regression QSARs for TTR binding. In another study [114], Yang et al. used the same approach as before and included the Tanimoto similarity index to assess the reliability of external predictions for four regression QSARs for TTR binding; in the same study, they combined the Euclidean distance-based method with the Tanimoto similarity index to define the AD of five classification QSARs. The Euclidean distance method was additionally employed to define the AD of two QSARs developed by Kar et al. [133] and one QSAR by Kovarich et al. [146]. Kar et al. [133] combined it with the standardisation-based technique [173], while Kovarich et al. [146] combined it with the range of descriptor values in the training set. Zhang et al. [87] employed the Hotelling T^2^ test to measure the distance of new compounds from the centre of the training set in descriptor space in order to define the AD. Rybacka and colleagues [137] used a PCA to define the chemical space of the training set based on selected molecular descriptors, and then calculated the distance-to-the-model (DModX) value for each compound. Methodologies less common than distance-based approaches were applied in six distinct publications [51,95,113,120,121,138]. Toropova et al. [138] defined the ADs of three QSARs according to the prevalence of local and global SMILES attributes in the training and validation sets, as proposed in their earlier publication [177]. Both Gadaleta et al. [120] and Rosenberg et al. [121] defined the ADs of their models in terms of the post probability of the predictions, with Rosenberg et al. [121] integrating the study of post probabilities with the Tanimoto similarity index. Liu et al. [95] characterised the AD in terms of weighted similarity density (ρ_s_) and weighted inconsistency of activities (I_A_) (AD_SAL_{ρs, IA}). Finally, both Dracheva et al. [51] and Sapounidou et al. [113] employed the CP framework to define the AD. As described in the studies, the CP quantifies the uncertainty of predictions by providing similarity scores, also termed as nonconformity scores, which can then be used to determine whether query compounds fall inside or outside the AD of a model.

Overall, a critical finding was the pronounced lack of QSAR models associated with a clearly defined AD. A clear definition of the AD is fundamental to increase confidence in the reliability of QSAR predictions and to accurately assess the degree of extrapolations. Without a defined AD, QSAR models risk being applied incorrectly and outside their intended scope, which can lead to the misuse of the tool and, ultimately, unreliable predictions.

### 3.8. Molecular Descriptors: Mechanistic Interpretations and Feature Importance

Molecular descriptors encode for numerical representations of molecular structures and serve as independent variables in QSAR models. Thousands of molecular descriptors have been developed, reflecting the varied complexity of chemical structural representation. Molecular descriptors range from simple molecular properties (e.g., molecular weight (MW)) to highly complex ones (e.g., quantum chemical descriptors) [178]. Multiple types of software, either open or commercial, are available for their calculation [179].

Across the examined studies, an extensive range of molecular descriptors and software for their calculation was observed. The full list of software and molecular descriptors used for each model is provided in Table 2, where models are presented for each MIE to facilitate direct comparison. These descriptors encompassed multiple categories, including physicochemical properties, FPs, constitutional, topological, electronic, and quantum chemical descriptors. It was a common practice to combine multiple software or libraries within a single study to compute different types of molecular descriptors.

The mechanistic interpretation of a QSAR model is critically important because it allows for the identification of the chemical properties or structural features that most significantly contribute to the predicted endpoint, enhancing the scientific credibility and acceptance of predictions [157,158]. Furthermore, it can offer new insights into the molecular features driving the modelled activity, hence contributing to safe-by-design approach. However, mechanistic interpretation is not always straightforward. This is often due to the challenging interpretability of certain molecular descriptors or the complexity of the algorithms used in model development [184]. To overcome this, feature importance techniques are often employed to provide clarity and to pinpoint the most influential molecular descriptors among many, since not all descriptors contribute equally.

Mechanistic interpretation or the application of feature importance techniques was conducted for fifty-six QSARs selected in this review, accounting for approximately 65% of the total models. These approaches were applied across six specific MIEs: TTR, TR, TSHR, TPO, TBG, and albumin. The decision to conduct a straightforward mechanistic interpretation of selected molecular descriptors or to apply feature importance techniques was contingent upon various factors, including the type of modelling methodology employed, the chemical nature of the compounds modelled, and the specific types of molecular descriptors used. Relevant descriptors are influenced by the structural characteristics included in the dataset, which in turn depends on whether the dataset is composed of structurally heterogeneous chemicals or of compounds from a single chemical class.

Interpreted QSARs for TTR binding were either based on heterogenous organic chemicals or specific chemical classes, including halogenated phenols and thiophenols, PFAS and/or PFCs, and PBDEs and their hydroxylated metabolites. A strong consensus on the fundamental molecular properties influencing TTR binding was revealed, although different descriptors were used to represent those properties, highlighting the fact that various computational methods can effectively encode the same critical structural information. The most significant and consistently identified structural features were aromatic rings, halogen atoms, and hydroxyl groups. Examples of descriptors encoding for these features were nArOH (number of aromatic hydroxyls) and nX (number of halogen atoms), which consistently showed a positive correlation with TTR binding affinity. These can be referred to as “structural alerts”, as their presence recalls the chemical structure of THs like T3 and T4. In addition, hydrophobicity was consistently recognised as a critical property driving TTR binding. Descriptors encoding for this property, such as logP and log DOW (pH = 7.40), were repeatedly selected in various QSARs. The hydrophobic nature of the TTR binding site for T4 justifies this observation [185]. Furthermore, descriptors like a_don, nHDon, and H-050 were selected to encode for hydrogen bond donor capacity, thereby emphasising the role of noncovalent interactions, such as hydrogen bonding and electrostatic interactions, between ligands and TTR. Furthermore, a consensus on the most significant features determining the TTR binding by PFAS (or PFCs) was highlighted across studies addressing this class of chemicals. These were mainly represented by the carbon chain length, MW and dimension, and terminal functional groups. An intermediate carbon chain length was found to be optimal for TTR binding. This information was encoded by descriptors like HATS6m and F06[C-O]. The most active PFAS were found to have an MW between 300 and 500 g/mol, as captured by the AMW descriptor. HATS6m, which encodes for molecular shape and dimension, was used to distinguish the activity of compounds with similar molecular weights. F07[C-O] and nH were used to account for the presence of carboxylic or sulfonic acid terminal groups at a particular topological distance and to differentiate compounds based on their terminal functional group. As seen before, hydrophobicity was still recognised as a critical property driving TTR binding. A broad spectrum of molecular descriptors was used across these studies, encompassing quantum chemical and electronic descriptors, topological, structural, and constitutional ones, as well as functional group counts and logKOW. It was often observed that the same groups of descriptors were employed across different studies, especially when conducted by the same research groups. While all studies converged on similar key features for TTR binding, it is worth noting that the specific choice and subsequent interpretation of descriptors could be influenced by a research group’s preferred modelling tools, their expertise, and their background. This implies that while the underlying findings may be consistent, their description might vary in the level of detail, depending on the specific approach adopted by the group.

As seen for TTR, interpreted QSARs for TR binding predictions were either based on heterogenous organic chemicals or specific classes. These included PCBs and their hydroxylated metabolites, PBDEs, PCNs, and PFAS. Similarly to TTR, TR modelling was performed using a wide array of molecular descriptors, and mechanistic interpretations were either more general or detailed. The presence and quantity of halogen atoms were consistently identified as being critical for TR binding. This information was encoded in descriptors like X% (percentage of halogen atoms) and nBr or nCl (number of bromine or chlorine atoms, respectively), which showed a positive correlation with TR binding affinity. In addition, molecular polarity was identified as a relevant property. Descriptors like EEig03d and EEig06d (edge adjacency indices weighted by dipole moments) and μ and μ2 (dipole moments) were positively correlated with TR binding, indicating that an increase in polarity could enhance affinity to TR. For PFAS, an optimal chain length was identified as a key determinant of TR binding, showing a moderate to high probability of binding for longer chains. Hydrophobicity was another key property consistently identified as a positive contributor to TR binding. Finally, electronic descriptors were selected to encode for the ability of a compound to accept or donate electrons and form hydrogen bonds with TR.

The interpretation of five QSARs for TPO inhibition by Seo et al. [107], Gadaleta et al. [120], and Rosenberg et al. [121] led to converged results, despite the use of different software to calculate molecular descriptors and different types of descriptors. The presence of aromatic structures, either hydroxylated (e.g., phenols) or non-hydroxylated (e.g., anilines), and of various heteroatoms (including nitrogen, oxygen, sulphur, and halogens) were highlighted as key structural features for TPO inhibition. These findings pointed out how these structural features often mimic typical endogenous targets of TPO, like tyrosine residues, thereby exerting disrupting effects [120]. Additionally, the lipophilic nature of a compound was also identified as a critical property. Furthermore, valuable insights regarding typical structural features found in non-TPO inhibitors were provided [121], including ethers, esters, aryl halides, and tertiary amines. All of these findings offered a comprehensive picture of which structural features and/or properties either contribute to, or detract from, TPO inhibition.

Regarding TSHR, two QSARs by Liu et al. [95] and Xu et al. [106] were interpreted. According to Xu et al. [106], the inhibitory effect of compounds on the TSHR is primarily influenced by two key chemical descriptors: the probability of water solubility (encoded by the descriptor “Sw < 0.1 mg/mL probability”) and lipophilicity (encoded by the descriptor “log D (pH = 7.4)”). The probability of water solubility was identified as the most influential factor because compounds must be able to diffuse through blood or body fluids to reach their biological target. Compounds with very low water solubility are less likely to be transported effectively, thus limiting their TSHR inhibitory potential. High lipophilicity was highlighted as key for TSHR inhibition, since this property describes the ability of compounds to penetrate the cell membrane and reach the transmembrane domain of TSHR. Nevertheless, several other molecular descriptors, reported in Table 2, were considered to account for factors influencing properties including dissociation properties, molecular flexibility, and electronic interactions. Liu et al. [95] employed the Shapley additive explanation (SHAP) technique to quantitatively assess the influence of each molecular feature, encoded as FPs, on TSHR agonism. While twenty different FPs were identified as having positive SHAP values, this analysis pointed out the contributions of lipophilicity, and aromatic and/or amino groups in promoting TSHR agonism.

TBG binding was only modelled by Yang et al., who developed four QSARs based on data for PBBs, including their mono-hydroxylated and di-hydroxylated metabolites [52]. The mechanistic interpretation indicated that hydroxylated metabolites exhibited a greater ability to bind with TBG, likely due to their capacity to establish hydrogen bonds or van der Waals interactions. Similarly, albumin binding was modelled only by Gallagher et al. [93], who developed three QSARs based on data for PFAS. Although they only provided indications about the positive or negative contributions of the selected molecular descriptors based on their coefficients signs, the authors concluded that PFAS with chain lengths shorter than ten carbons demonstrated a higher albumin binding affinity compared with those with longer carbon chains.

Overall, although these findings provided valuable insights into the main structural features and properties that may cause TH system disruption, a drawback is the limited emphasis or, in some instances, the complete absence of mechanistic interpretations or the application of feature importance techniques. This may limit the confidence in QSAR models among both scientists and regulatory bodies. Therefore, considering the increasing demand for mechanistically informed NAMs to advance chemical hazard assessments, future research should prioritise and dedicate resources to improving the mechanistic understanding of QSAR models in order to promote wider acceptance and trust in these methodologies.

Finally, this review showed the wide array of software and molecular descriptors used in QSAR studies for TH system disruption, underscoring the dynamic nature of the field. The diversity in approaches to descriptor calculation and selection indicates a lack of a single, standardised tool or method. Instead, the choice of software and methodology appeared to be driven by factors such as the expertise of researchers, tool accessibility, and prior experience with specific platforms.

### 3.9. Recent Advances: 2025

To keep this review up to date and to provide a picture of the field’s evolution, this section was included to provide a concise picture of the key models published between January and July 2025. Table 3 and Table 4 are included for quick reference.

Charest et al. [187] developed a QSAR model for TTR binding prediction using RF on a dataset of 853 compounds. The AD was defined using prediction entropy (PS), a metric derived from the probability outputs from the RF. A core strength of their modelling process was the adoption of a “mechanistic a priori” approach. They first analysed crystal structures of TTR and performed docking studies of how chemicals bind to it, allowing the authors to select molecular descriptors that were known to be relevant to the binding mechanism. The chosen descriptors, obtained from the PaDEL descriptor library using the OPERA software v2.9 [192], included measures of hydrogen bonding (nHBacc, nHBDon), planarity (naAromAtom), and hydrophobicity (CrippenLogP), as well as more complex topological descriptors like ETA and ATSC to capture fine structural details. They also included ZMIC descriptors to account for the structural specificity of protein binding. After training the model, they used the permutation importance and the mutual information methods to perform an a posteriori analysis to confirm the importance of their selected features. The authors found that the descriptors related to aromatic structures and hydrophobicity were highly relevant for TTR binding, which aligned with their initial mechanistic hypothesis and were consistent with mechanistic interpretations by previous studies described in Section 3.8. This combination of a priori and a posteriori analysis turned the model from a “black box” into a transparent and interpretable tool that is beyond the sole generation of predictions. Janicka et al. [186] developed a QSAR model designed to predict albumin binding based on data for twenty-nine phenoxyacetic acid-derived congeners. The authors first applied biopartitioning micellar chromatography (BMC) to derive an in vitro lipophilicity descriptor (logkBMC), which was used with other descriptors to develop an MLR-QSAR. The leverage approach, with the use of the Williams plot for graphical visualisation, was used to define the AD. Three key molecular descriptors defined the model: logkBMC to encode for lipophilicity, α to encode for polarizability, and the sum of hydrogen bond donors (HBD) and acceptors (HBA). Based on the descriptors’ signs in the model’s equation, binding to albumin was found to increase with higher lipophilicity and polarizability and to decrease with a greater number of hydrogen bond donors and acceptors. Two QSAR models were developed by Evangelista et al. [189] to predict the binding of PFAS to TTR, using a dataset of 134 PFAS. One classification model was developed using LDA, while one regression model was developed using MLR. To ensure robustness and avoid overfitting, the models were subjected to a rigorous validation protocol including randomization procedures and leave-one-out bootstrapping. The AD was defined differently for each model. For the classification model, the AD was defined in terms of distance (cosine α) and post probabilities of classification. Shannon entropy was introduced to quantify the uncertainty associated with external predictions. For the regression model, the AD was defined in terms of the leverage approach, with the adoption of the Williams plot for graphical visualisation. A prediction interval was introduced to quantify the uncertainty associated with external predictions. The classification model was characterised by GATS3e, ATSC6p, GATS8m, and MIC2, while the regression model was characterised by piPC5, GGI9, and AATSC0e. The selected descriptors were consistent with prior in vitro and in silico (docking) findings regarding the major drivers of PFAS binding to TTR. The findings highlighted the importance of hydrogen bond formation and hydrophobic interactions to establish binding with TTR. The relevance of lipophilicity, molecular weight, and chain length were highlighted. The study performed by Sosnowska et al. [191] focused on the ability of PFAS to bind to TTR. The methodology involved the development of classification and regression QSAR models based on data from 45 PFAS. The classification model was developed using the decision tree classifier (DTC). Then, a single regression model was developed using MLR. The same algorithm was also used to perform a multiple regression model (MRM) approach, where a total of thirty-one single MLR-QSARs were developed from different data splits. The AD for the classification model was defined using a boundary box method, while the leverage approach with the support of the Williams plot was employed to define the AD of the regression QSARs. The classification model highlighted molecular size and structural complexity as key factors, using descriptors like SM4_D and GATS3m. The MLR model emphasised that compounds with heavier and more polar atoms tended to be more active, with descriptors like AMW and GATS7p. It also identified the importance of specific structural features, such as fluorine atoms at a topological distance of 10 bonds (B10[F-F]). Three molecular descriptors were frequently selected in the models developed through the MRM approach (JGI10, ATSC7c, and MATS6i), which further confirmed the importance of atomic charge and polarity. The QSAR models generated via the MRM approach were not included in this review for simplicity because their performance was comparable to that of the single model developed independently. The collective findings consistently showed that molecular size, complexity, and polarity were the primary drivers of PFAS activity in disrupting TTR. Ultimately, it is highly relevant to cite the study by Cirino et al. [193]. Although their work did not propose any new QSAR models, its focus on optimising predictive performance through consensus modelling and evaluating the robustness of existing models made it a significant contribution to the field. Specifically, this study served as a retrospective analysis of computational strategies from the Tox24 Challenge [194], which aimed to advance computational toxicology for predicting chemical binding to TTR by using a large dataset of 1512 compounds [188]. The primary goal by Cirino et al. [193] was to analyse the models developed by the nine top-performing teams from the Tox24 Challenge and explore consensus strategies to enhance the predictive performances of single models. The participating teams adopted diverse strategies, with some relying on single-method models while others combined multiple approaches, such as descriptor-based and representation learning techniques. The study by Cirino et al. [193] demonstrated that consensus modelling improved the predictive accuracy for TTR binding, compared with individual models alone, achieving a lower error rate. Finally, an analysis to identify overrepresented functional groups in active compounds for TTR binding was performed. Unsurprisingly, groups similar to T4, such as phenols, aryl halides, and diarylethers, were highly frequent. Six other functional groups of potential concern were identified, including nitro compounds, arenes, and gem-trihalides.

## 4. Conclusions

This review highlighted the growing yet still-evolving landscape of QSAR models addressing MIEs leading to TH system disruption by chemical substances. While significant progress has been made, particularly due to the increased availability of HTS data, the field remains fragmented and challenges persist. This review highlighted a preference for classification-based models to predict categorical outcomes, instead of continuous toxicity values, and that, despite the rise in complex machine learning methods, simpler algorithms continued to be employed to leverage their interpretability and promote broader adoption.

This review revealed that modelling efforts were predominantly focused on key MIEs like TR and TTR, scarcely followed by TPO and TSHR. Critically, many other relevant MIEs, including the three deiodinases, NIS, TRHR, TBG, and albumin were significantly poorly addressed in QSAR research. A critical finding was the lack of QSAR modelling studies addressing MIEs related to DUOX, IYD, and pendrin inhibition, and those associated with cellular TH transport (specifically MCT8, MCT10, OATP1C1, OATP1A4, MDR1, and MRP2), highlighting critical areas for future investigations. Similarly, a limited number of chemical classes were addressed, leading to a very small number of local QSARs. Notably, while validation strategies were consistently employed, a critical finding was a frequent lack of explicitly defined ADs. Without clear AD definitions, QSARs risk being applied outside their scope, undermining decision-making confidence and leading to the incorrect use of QSARs. Furthermore, even though several types of molecular descriptors have been consistently identified as being relevant to model specific MIEs (e.g., TTR and TR), a limited emphasis on mechanistic interpretations was observed for many models, representing a critical drawback. However, the recent emergence of studies simultaneously covering multiple TH system-related endpoints demonstrated a growing awareness of the multifaceted nature of TH system disruption, offering a promising direction in aligning predictive modelling within AOP frameworks. The findings suggested a need for increased efforts in generating in vitro and in silico data for poorly addressed MIEs, broadening the chemical space of tested compounds, and ultimately developing new models. The successful application of integrated in silico approaches to generate activity data, such as molecular docking and dynamic simulations, has proven to be an effective strategy for developing QSARs when experimental data is limited or unavailable, presenting a valuable path forward for exploring multiple MIEs and for specific chemical classes. This would enable a more robust hazard assessment for entire groups of compounds. Future studies should prioritise the development of QSAR models with clearly defined ADs and enhanced mechanistic interpretability to increase the reliability and transparency of and confidence in their predictions, ultimately to promote their wider acceptance as effective NAMs for TH system disruption assessment.

## Figures and Tables

**Table 1:** Summary and main characteristics of selected QSARs. C: classification-based; R: regression-based; Primary: data generated as part of the same study; Secondary: data collected from the existing literature; ToxCast database: Toxicity Forecaster (ToxCast) database (https://www.epa.gov/comptox-tools/toxicity-forecasting-toxcast); Tox21 database: Toxicology in the 21st Century (Tox21) (https://tox21.gov/); Ref.: reference; n.s.: not specified.

Model ID	Ref.	Year	MIE	Algorithm	C or R	Chemical Class	Data Source Type	Data Source Literature Reference(s)
ID_1	[52]	2024	TBG	MLR	R	PBBs	Primary	[52]
ID_2	[52]	2024	TBG	MLR	R	PBBs and OH-PBBs	Primary	[52]
ID_3	[52]	2024	TBG	MLR	R	PBBs and 2OH-PBBs	Primary	[52]
ID_4	[52]	2024	TBG	MLR	R	PBBs, OH-PBBs, and 2OH-PBBs	Primary	[52]
ID_5	[53]	2024	TTR	MLR	R	Heterogeneous	Secondary	[54,55,56,57,58,59,60]
ID_6	[53]	2024	TTR	MLR	R	Heterogeneous	Secondary	[61,62,63,64,65,66,67]
ID_7	[53]	2024	TTR	MLR	R	Heterogeneous	Secondary	[68,69,70,71,72,73,74,75,76,77,78,79,80,81,82,83,84,85,86,87,88,89]
ID_8	[90]	2023	TR α	MLR	R	PFAS	Primary	[90]
ID_9	[90]	2023	TR β	MLR	R	PFAS	Primary	[90]
ID_10	[91]	2023	TR n.s.	LDA	C	OH-PCBs	Secondary	[92]
ID_11	[91]	2023	TR n.s.	LR	C	OH-PCBs	Secondary	[92]
ID_12	[93]	2023	Albumin	PLS	R	PFAS	Secondary	[94]
ID_13	[93]	2023	Albumin	LDA	C	PFAS	Secondary	[94]
ID_14	[93]	2023	Albumin	MLR	R	PFAS	Secondary	[94]
ID_15	[95]	2023	TSHR	RF	C	Heterogeneous	Tox21 database and secondary	[96,97,98]
ID_16	[51]	2022	TTR	RF	C	Heterogeneous	Secondary	[87]
ID_17	[51]	2022	TR β	RF	C	Heterogeneous	Tox21 database *	[99,100]
ID_18	[51]	2022	TR β	RF	C	Heterogeneous	Tox21 database *	[99,100]
ID_19	[51]	2022	TSHR	RF	C	Heterogeneous	Tox21 database *	[99,100]
ID_20	[51]	2022	TSHR	RF	C	Heterogeneous	Tox21 database *	[99,100]
ID_21	[51]	2022	TRHR	RF	C	Heterogeneous	Tox21 database *	[99,100]
ID_22	[51]	2022	DIO1	RF	C	Heterogeneous	ToxCast database **	[101]
ID_23	[51]	2022	DIO2	RF	C	Heterogeneous	ToxCast database **	[101]
ID_24	[51]	2022	DIO3	RF	C	Heterogeneous	ToxCast database **	[101]
ID_25	[51]	2022	NIS	RF	C	Heterogeneous	ToxCast database **	[102]
ID_26	[51]	2022	TPO	RF	C	Heterogeneous	ToxCast database **	[103]
ID_27	[104]	2022	TTR	RF	C	Heterogeneous	ChEMBL database ***	[105]
ID_28	[104]	2022	TR α	RF	C	Heterogeneous	ChEMBL database ***	[105]
ID_29	[104]	2022	TR β	RF	C	Heterogeneous	ChEMBL database ***	[105]
ID_30	[104]	2022	NIS	RF	C	Heterogeneous	ChEMBL database ***	[105]
ID_31	[106]	2022	TSHR	RF	C	Heterogeneous	Tox21 database	https://tripod.nih.gov/tox21/assays/
ID_32	[106]	2022	TSHR	RF	C	Heterogeneous	Tox21 database	https://tripod.nih.gov/tox21/assays/
ID_33	[106]	2022	TSHR	XGB	C	Heterogeneous	Tox21 database	https://tripod.nih.gov/tox21/assays/
ID_34	[106]	2022	TSHR	LR	C	Heterogeneous	Tox21 database	https://tripod.nih.gov/tox21/assays/
ID_35	[106]	2022	TSHR	XGB	R	Heterogeneous	Tox21 database	https://tripod.nih.gov/tox21/assays/
ID_36	[107]	2022	TPO	XGB	C	Heterogeneous	ToxCast database and secondary **	[103,108,109,110,111]
ID_37	[107]	2022	TPO	Hard Voting	C	Heterogeneous	ToxCast database and secondary **	[103,108,109,110,111]
ID_38	[107]	2022	TPO	Soft Voting	C	Heterogeneous	ToxCast database and secondary **	[103,108,109,110,111]
ID_39	[112]	2022	TR β	MLR	R	PCNs	Primary	[112]
ID_40	[113]	2023	TR β	RF	C	Heterogeneous	Tox21 database	National Center for Biotechnology Information. PubChem Database. Source = 824, AID = 743067, https://pubchem.ncbi.nlm.nih.gov/bioassay/743067 (accessed 13 May 2021)
ID_41	[59]	2021	TTR	MLR	R	Halogenated phenols and thiophenols	Primary and Secondary	[57,59]
ID_42	[114]	2021	TTR	kNN	C	Heterogeneous	Secondary	[54,55,56,57,58,59,61,62,64,68,70,71,72,73,75,78,79,80,81,82,83,84,85,86,87,88,89,115,116,117,118]
ID_43	[114]	2021	TTR	kNN	C	Heterogeneous	Secondary	[54,55,56,57,58,59,61,62,64,68,70,71,72,73,75,78,79,80,81,82,83,84,85,86,87,88,89,115,116,117,118]
ID_44	[114]	2021	TTR	kNN	C	Heterogeneous	Secondary	[54,55,56,57,58,59,61,62,64,68,70,71,72,73,75,78,79,80,81,82,83,84,85,86,87,88,89,115,116,117,118]
ID_45	[114]	2021	TTR	kNN	C	Heterogeneous	Secondary	[54,55,56,57,58,59,61,62,64,68,70,71,72,73,75,78,79,80,81,82,83,84,85,86,87,88,89,115,116,117,118]
ID_46	[114]	2021	TTR	kNN	C	Heterogeneous	Secondary	[54,55,56,57,58,59,61,62,64,68,70,71,72,73,75,78,79,80,81,82,83,84,85,86,87,88,89,115,116,117,118]
ID_47	[114]	2021	TTR	MLR	R	Heterogeneous	Secondary	[61,70,71,72,73,75,78,79,80,81,82,83,84,85,86,87,88]
ID_48	[114]	2021	TTR	MLR	R	Heterogeneous	Secondary	[57,58,59]
ID_49	[114]	2021	TTR	kNN	R	Heterogeneous	Secondary	[61,70,71,72,73,75,78,79,80,81,82,83,84,85,86,87,88]
ID_50	[114]	2021	TTR	kNN	R	Heterogeneous	Secondary	[57,58,59]
ID_51	[119]	2021	TR n.s.	RF	C	Heterogeneous	ToxCast database	Cited as ToxCast and Tox21 Summary Files for invitroDBv3.2, U.S. EPA, Washington, DC.
ID_52	[119]	2021	TSHR	RF	C	Heterogeneous	ToxCast database	Cited as ToxCast and Tox21 Summary Files for invitroDBv3.2, U.S. EPA, Washington, DC.
ID_53	[119]	2021	TSHR	NN	C	Heterogeneous	ToxCast database	Cited as ToxCast and Tox21 Summary Files for invitroDBv3.2, U.S. EPA, Washington, DC.
ID_54	[119]	2021	TPO	XGB	C	Heterogeneous	ToxCast database **	Cited as ToxCast and Tox21 Summary Files for invitroDBv3.2, U.S. EPA, Washington, DC. and [103]
ID_55	[119]	2021	TRHR	SVM	C	Heterogeneous	ToxCast database	Cited as ToxCast and Tox21 Summary Files for invitroDBv3.2, U.S. EPA, Washington, DC.
ID_56	[119]	2021	DIO1	SVM	C	Heterogeneous	ToxCast database **	Cited as ToxCast and Tox21 Summary Files for invitroDBv3.2, U.S. EPA, Washington, DC. and [101]
ID_57	[119]	2021	DIO2	SVM	C	Heterogeneous	ToxCast database **	[101]
ID_58	[119]	2021	DIO3	NN	C	Heterogeneous	ToxCast database **	[101]
ID_59	[119]	2021	NIS	LR	C	Heterogeneous	ToxCast database **	Cited as ToxCast and Tox21 Summary Files for invitroDBv3.2, U.S. EPA, Washington, DC. and [102]
ID_60	[120]	2021	TPO	kNN	C	Heterogeneous	ToxCast database **	[103,121]
ID_61	[120]	2021	TPO	RF	C	Heterogeneous	ToxCast database **	[103,121]
ID_62	[57]	2019	TTR	MLR	R	Phenolic DBPs	Primary	[57]
ID_63	[122]	2018	TR β	SVM	C	PCBs	Primary	[122]
ID_64	[122]	2018	TR β	LDA	C	PCBs	Primary	[122]
ID_65	[123]	2018	TR n.s.	SVM	C	PCBs and PBDEs	Secondary	[124,125,126,127,128,129,130,131,132]
ID_66	[133]	2017	TTR	LDA	C	PFCs	Secondary	[82]
ID_67	[133]	2017	TTR	MLR	R	PFCs	Secondary	[82]
ID_68	[121]	2017	TPO	PLR	C	Heterogeneous	ToxCast database **	[103,134,135,136]
ID_69	[121]	2017	TPO	PLR	C	Heterogeneous	ToxCast database **	[103,134,135,136]
ID_70	[87]	2015	TTR	kNN	C	Heterogeneous	Secondary	[88]
ID_71	[137]	2015	TTR	ASNN	C	Heterogeneous	Secondary	[88]
ID_72	[138]	2015	TR β	Monte Carlo	R	Heterogeneous	Secondary	[139]
ID_73	[138]	2015	TR β	Monte Carlo	R	Heterogeneous	Secondary	[139]
ID_74	[138]	2015	TR β	Monte Carlo	R	Heterogeneous	Secondary	[139]
ID_75	[139]	2014	TR β	RF	R	Heterogeneous	ChEMBL database ***	[140]
ID_76	[139]	2014	TR β	RF	R	Heterogeneous	Secondary	[141,142,143]
ID_77	[139]	2014	TR β	RF	C	Heterogeneous	ChEMBL database ***	[140]
ID_78	[144]	2013	TTR	kNN	C	PFCs and BFRs	Secondary	[78,80,82]
ID_79	[144]	2013	TTR	MLR	R	PFCs and BFRs	Secondary	[78,80,82]
ID_80	[145]	2012	TTR	kNN	C	PFCs	Secondary	[82]
ID_81	[145]	2012	TTR	kNN	C	PFCs	Secondary	[82]
ID_82	[145]	2012	TTR	kNN	C	PFCs	Secondary	[82]
ID_83	[145]	2012	TTR	kNN	C	PFCs	Secondary	[82]
ID_84	[146]	2011	TTR	kNN	C	BFRs	Secondary	[78,80]
ID_85	[147]	2010	TTR	MLR	R	BFRs	Secondary	[78,80]
ID_86	[148]	2010	TR β	PLS	R	OH-PBDEs	Primary	[148]

* Tox21 served as a data source but it was cited as [99,100]. ** Although cited as [101,102,103] or [134,135,136], this review will refer to them as the ToxCast data source as described in the referenced papers. *** ChEMBL served as a data source but it was cited as [105] or [140].

**Table 2:** Summary of the molecular descriptors selected by each QSAR, grouped by MIE.

MIE	Ref.	Model ID	Chemical Class	Descriptors	Software
TTR	[53]	ID_5	Heterogeneous	AATSC1c; PubchemFP381; ATSC2s; nX	PaDEL [180]
		ID_6	Heterogeneous	naasC; SpMin4_Bhs; VE3_Dzs	PaDEL [180]
		ID_7	Heterogeneous	PubchemFP590; SpMax1_Bhe; PubchemFP18; GATS5c; AATSC1e; AATS4v	PaDEL [180]
	[51]	ID_16	Heterogeneous	Calculation of 119 RDKit chemical descriptors	RDKit: Open-source cheminformatics. http://www.rdkit.org
	[104]	ID_27	Heterogeneous	Calculation of extended fingerprints with a KNIME implementation of the CDK toolkit	CDK toolkit: https://cdk.github.io/
	[59]	ID_41	Halogenated phenols and thiophenols	logDOW(pH = 7.40); ω_adj_; dipole_adj_	Marvin Sketch 15.6.29.0, 2015: ChemAxon, http://www.chemaxon.com); Gaussian 16; GsGrid 1.7 (http://gsgrid.codeplex.com)
	[114]	ID_42	Heterogeneous	V_sadj_; Π_adj_; μ_adj_	Marvin Sketch 15.6.29.0, 2015: ChemAxon, http://www.chemaxon.com; GaussView 6.0; Gaussian 16; GsGrid 1.7, http://gsgrid.codeplex.com
		ID_43	Heterogeneous	V_sadj_; O-059; μ_adj_	Marvin Sketch 15.6.29.0, 2015: ChemAxon, http://www.chemaxon.com; GaussView 6.0; Gaussian 16; GsGrid 1.7, http://gsgrid.codeplex.com
		ID_44	Heterogeneous	V_sadj_; H-050; nCbH	Marvin Sketch 15.6.29.0, 2015: ChemAxon, http://www.chemaxon.com; GaussView 6.0; Gaussian 16; GsGrid 1.7, http://gsgrid.codeplex.com
		ID_45	Heterogeneous	nArOH; V_sadj_; ω_adj_	Marvin Sketch 15.6.29.0, 2015: ChemAxon, http://www.chemaxon.com; GaussView 6.0; Gaussian 16; GsGrid 1.7, http://gsgrid.codeplex.com
		ID_46	Heterogeneous	V_sadj_; C-024; nHDon	Marvin Sketch 15.6.29.0, 2015: ChemAxon, http://www.chemaxon.com; GaussView 6.0; Gaussian 16; GsGrid 1.7, http://gsgrid.codeplex.com
		ID_47	Heterogeneous	C-040; nCq; H-050; O-058; Πadj; O-056	Marvin Sketch 15.6.29.0, 2015: ChemAxon, http://www.chemaxon.com; GaussView 6.0; Gaussian 16; GsGrid 1.7, http://gsgrid.codeplex.com
		ID_48	Heterogeneous	log DOW(pH = 7.40); nArOH; O-057; nArNO2	Marvin Sketch 15.6.29.0, 2015: ChemAxon, http://www.chemaxon.com; GaussView 6.0; Gaussian 16; GsGrid 1.7, http://gsgrid.codeplex.com
		ID_49	Heterogeneous	E_HOMO-adj_; nArOH; H052; ω_adj_	Marvin Sketch 15.6.29.0, 2015: ChemAxon, http://www.chemaxon.com; GaussView 6.0; Gaussian 16; GsGrid 1.7, http://gsgrid.codeplex.com
		ID_50	Heterogeneous	log DOW(pH = 7.40); nArOH	Marvin Sketch 15.6.29.0, 2015: ChemAxon, http://www.chemaxon.com; GaussView 6.0; Gaussian 16; GsGrid 1.7, http://gsgrid.codeplex.com
	[57]	ID_62	Phenolic DBPs	log D; dipole_adj_	Marvin Sketch 15.6.29.0, 2015: ChemAxon, http://www.chemaxon.com; Gaussian 16
	[133]	ID_66	PFCs	Me; nCsp2; H-050	DRAGON Version 6.0, 2011, http://www.talete.mi.it/
		ID_67	PFCs	IC3; ∑β’_S_	DRAGON Version 6.0, 2011, http://www.talete.mi.it/
	[87]	ID_70	Heterogeneous	Based on the following 14 molecular descriptors: TPSA; a_don; a_nOH; nX; PEOE_VSA_FNEG; PEOE_RPC-; density; PEOE_RPC+; diameter; PEOE_PC+; vsa_hyd; KierFlex; logP(o/w); opr_brigid	Molecular Operating Environment (MOE), 2013.08; Chemical Computing Group Inc.: Montreal, QC, Canada, 2015
	[137]	ID_71	Heterogeneous	nArOH; nHDon; nCb-; nCRX3; nCH2RX; ALogPS_logP; nArOR; nCrq; nCq; nCp; nCs; nCbH	DRAGON version 6 [181].
	[144]	ID_78	PFCs and BFRs	nArOH; F03(Br..Br); HATS6m	DRAGON Version 5.5 for Windows, Talete srl, Milan, Italy, 2007
		ID_79	PFCs and BFRs	R5u; F07[C-O]; nArOH	DRAGON Version 5.5 for Windows, Talete srl, Milan, Italy, 2007
	[145]	ID_80	PFCs	AMW; HATS6m	DRAGON Version 5.5 for Windows, Talete srl, Milan, Italy, 2007
		ID_81	PFCs	nH; HATS6m	DRAGON Version 5.5 for Windows, Talete srl, Milan, Italy, 2007
		ID_82	PFCs	nH; F06[C-O]	DRAGON Version 5.5 for Windows, Talete srl, Milan, Italy, 2007
		ID_83	PFCs	T(F..F); HATS6m	DRAGON Version 5.5 for Windows, Talete srl, Milan, Italy, 2007
	[146]	ID_84	BFRs	DISPe; nArOH	DRAGON Version 5.5 for Windows, Talete srl, Milan, Italy, 2008
	[147]	ID_85	BFRs	qpmax; MATS6v	DRAGON Version 5.5 for Windows, Talete srl, Milan, Italy
TR α	[90]	ID_8	PFAS	X%; ICR	AlvaDesc [182]
	[104]	ID_28	Heterogeneous	Calculation of extended fingerprints with a KNIME implementation of the CDK toolkit	CDK toolkit: https://cdk.github.io/
TR β	[90]	ID_9	PFAS	X%; TPC	AlvaDesc [182]
	[51]	ID_17	Heterogeneous	Calculation of 119 RDKit chemical descriptors	RDKit: Open-source cheminformatics. http://www.rdkit.org
		ID_18	Heterogeneous	Calculation of 119 RDKit chemical descriptors	RDKit: Open-source cheminformatics. http://www.rdkit.org
	[104]	ID_29	Heterogeneous	Calculation of extended fingerprints with a KNIME implementation of the CDK toolkit	CDK toolkit: https://cdk.github.io/
	[112]	ID_39	PCNs	E_LUMO_; ΔE; μ; Q_xx_; Q_yy_; Q_yz_; q^+^; logK_ow_; N_Cl_; N_o_	Gaussian 09 software.
	[113]	ID_40	Heterogeneous	Use of RDKit descriptors	RDKit: Open-source cheminformatics. http://www.rdkit.org
	[122]	ID_63	PCBs	logK_ow_; ω; BER; nCl; EEig13d; JGI4	EPI Suite, version 4.1 (US EPA, 2012); DRAGON
		ID_64	PCBs	logK_ow_; ω; BER; nCl; EEig13d; JGI4	EPI Suite, version 4.1 (US EPA, 2012); DRAGON
	[138]	ID_72	Heterogeneous	Molecular optimal descriptor DCW(3, 10)	CORAL software: http://www.insilico.eu/coral
		ID_73	Heterogeneous	Molecular optimal descriptor DCW(1, 3)	CORAL software: http://www.insilico.eu/coral
		ID_74	Heterogeneous	Molecular optimal descriptor DCW(3, 4)	CORAL software: http://www.insilico.eu/coral
	[139]	ID_75	Heterogeneous	Thirty-five most statistically significant descriptors were identified: F04[N-Cl]; EEig03d; F06[C-Cl]; EEig08r; GATS7e; nArOH; EEig07r; EEig05d; EEig06d; TPSA(Tot); GGI1; BEHp4; SPI; C-026; ESpm01d; nCb-; Hy; GATS8v; T(O..O); BLTA96; IVDE; MATS1e; Ms; GATS6e; MATS6m; MATS5m; MATS2e; MATS1p; MATS8v; MATS6e; MATS8p; X4Av; X2Av; X0Av; Jhetp	Dragon software (version 5.4; Talete s.r.l., Milan, Italy)
		ID_76	Heterogeneous	Twenty-seven most statistically significant descriptors were identified: F08[C-Cl]; T(N..Cl); C-006; EEig06d; SEigm; ATS3m; ATS4m; BEHm6; T(O..Cl); ATS5m; ATS7m; BEHm7; Uindex; EEig04d; BELe3; EEig08d; HVcpx; PHI; BELm3; GGI8; BIC5; BEHml; JGI6; JGI7; BELml; GATS3p; VEA2	Dragon software (version 5.4; Talete s.r.l., Milan, Italy)
		ID_77	Heterogeneous	Thirty most statistically significant descriptors were identified: B05[O-O]; EEig03d; nArOH; GGI7; EEig05d; PW2; F04[C-N]; C-026; ESpm01d; AAC; GATS8p; Hy; PCR; GATS8v; F05[O-O]; O-057; MATS5v; IVDE; MATS1e; Ms; MATS5p; ARR; MATS5m; PHI; MATS8v; GATS1e; MATS8p; RBF; Jhetp; X1A	Dragon software (version 5.4; Talete s.r.l., Milan, Italy)
	[148]	ID_86	OH-PBDEs	nBr; logKow; I_A_; E_LUMO_; ω; μ^2^	EPI Suite, version 4.0 (U.S. Environmental Protection Agency 2009); Gaussian 03 programs; DRAGON [181]
TR n.s.	[91]	ID_10	OH-PCBs	RDF35u; RDF55u; RDF85u; RDF65v	PaDEL [180]
		ID_11	OH-PCBs	RDF35u; RDF55u; RDF85u; RDF65v	PaDEL [180]
	[119]	ID_51	Heterogeneous	Calculation of count-based Morgan fingerprints with a radius of 2 bonds and a length of 2048 bits, and of all 119 one-dimensional and two-dimensional RDKit chemical descriptors	RDKit: Open-source cheminformatics. http://www.rdkit.org
	[123]	ID_65	PCBs; PBDEs	DELS; MAXDN; Mor31v; Ms; RDF040e; BER	DRAGON 5.5 for Windows, Talete srl, Milan, Italy, 2008
TSHR	[106]	ID_31	Heterogeneous	Thirty-nine descriptors were used, here sorted by their weight in descending order (top seven descriptors were used to build Model ID_32.): Sw < 0.1 mg/mL probability; LogSw; LogD(pH = 7.4); LogL; S; R2; E; LogS(pH = 7.4); logP; Solubility class; AAB/LogP; McGowan Volume; MW; Pi2; LogS(pH = 7.4)-; L; V; Sw < 1 mg/mL probability; No Of H Donors; Acid_pKa; LogSwLo; Sw > 10 mg/mL probability; Abraham’s Alfa; NoOfRotBonds; A; Bo; 0Form; B; Form+; No Of H Acceptors; LogSwHi; Rel_pKa_ac; Base_pKa; Abraham’s BetaH; Ertl TPSA; Form-; Rule of 5; Rel_pKa_bs; Form±	KOWWIN program (EPI Suite version 4.1.1, https://www.epa.gov/tsca-screening-tools/epi-suitetm-estimation-program-interface) to calculate logKow. Software for the calculation of the other molecular descriptors was not specified
		ID_32		Sw < 0.1 mg/mL probability; LogSw; LogD(pH = 7.4); LogL; S; R2; E	KOWWIN program (EPI Suite version 4.1.1, https://www.epa.gov/tsca-screening-tools/epi-suitetm-estimation-program-interface) to calculate logKow. Software for the calculation of the other molecular descriptors was not specified
		ID_33		The use of thirty-nine descriptors was reported in the study	KOWWIN program (EPI Suite version 4.1.1, https://www.epa.gov/tsca-screening-tools/epi-suitetm-estimation-program-interface) to calculate logKow. Software for the calculation of the other molecular descriptors was not specified
		ID_34		LogS, LogP, E	KOWWIN program (EPI Suite version 4.1.1, https://www.epa.gov/tsca-screening-tools/epi-suitetm-estimation-program-interface) to calculate logKow. Software for the calculation of the other molecular descriptors was not specified
		ID_35		Forty-one descriptors were used, here sorted by their weight in descending order: Base_pKa; V; Abraham’s Alfa; 0Form; AAB/LogP; CDocker Energy; NoOfRotBonds; S; LogSwLo; LogSwHi; CDocker Interaction Energy; Rel_pKa_bs; R2; E; LogD(pH = 7.4); LogS(pH = 7.4)-; Sw < 0.1 mg/mL probability; A; Sw > 10 mg/mL probability; Ertl TPSA; MW; logP; LogSw; Pi2; Abraham’s BetaH; Solubility class; B; LogL; Sw < 1 mg/mL probability; L; Acid_pKa; Rel_pKa_ac; No Of H Acceptors; Bo; No Of H Donors; McGowan Volume; LogS(pH = 7.4); Form+; Form-; Form±; Rule of 5	KOWWIN program (EPI Suite version 4.1.1, https://www.epa.gov/tsca-screening-tools/epi-suitetm-estimation-program-interface) to calculate logKow. Software for the calculation of the other molecular descriptors was not specified
	[51]	ID_19	Heterogeneous	Calculation of 119 RDKit chemical descriptors	RDKit: Open-source cheminformatics. http://www.rdkit.org
		ID_20	Heterogeneous	Calculation of 119 RDKit chemical descriptors	RDKit: Open-source cheminformatics. http://www.rdkit.org
	[119]	ID_52	Heterogeneous	Calculation of count-based Morgan fingerprints with a radius of 2 bonds and a length of 2048 bits, and of all 119 one-dimensional and two-dimensional RDKit chemical descriptors	RDKit: Open-source cheminformatics. http://www.rdkit.org
		ID_53	Heterogeneous	Calculation of count-based Morgan fingerprints with a radius of 2 bonds and a length of 2048 bits, and of all 119 one-dimensional and two-dimensional RDKit chemical descriptors	RDKit: Open-source cheminformatics. http://www.rdkit.org
	[95]	ID_15	Heterogeneous	Top twenty FPs with positive SHAP (Shapley additive explanation) values: PubchemFP12, PubchemFP259, PubchemFP257, PubchemFP256, PubchemFP628, PubchemFP185, PubchemFP258, PubchemFP2, PubchemFP143, PubchemFP146, PubchemFP656, PubchemFP633, PubchemFP150, PubchemFP464, PubchemFP442, PubchemFP607, PubchemFP613, PubchemFP549, PubchemFP153, PubchemFP418	PaDEL [180]
TPO	[51]	ID_26	Heterogeneous	Calculation of 119 RDKit chemical descriptors	RDKit: Open-source cheminformatics. http://www.rdkit.org
	[107]	ID_36	Heterogeneous	Use of Atom Pair Count (APC) fingerprints	PaDEL [180]
		ID_37	Heterogeneous	Use of Atom Pair Count (APC) fingerprints	PaDEL [180]
		ID_38	Heterogeneous	Use of Atom Pair Count (APC) fingerprints	PaDEL [180]
	[119]	ID_54	Heterogeneous	Calculation of count-based Morgan fingerprints with a radius of 2 bonds and a length of 2048 bits, and of all 119 one-dimensional and two-dimensional RDKit chemical descriptors	RDKit: Open-source cheminformatics. http://www.rdkit.org
	[120]	ID_60	Heterogeneous	The top twenty ranked descriptors identified in the kNN model: GATS1e; NArOH; CATS2D_02_DL; MATS1e; MATS1s; C-026; CATS2D_03_DL; B10 [C-C]; MATS1m; ‘SpMax2_Bh(s); MATS1p; nCb-; NX; Uc; ‘P_VSA_i_1’; SpMAD_B(v); NCbH; GATS1s; MLOGP; Eta_C_A’	DRAGON v7.0.8., 2017: https://chm.kode-solutions.net/products_dragon.php
		ID_61	Heterogeneous	Based on 160 molecular descriptors	DRAGON v7.0.8., 2017: https://chm.kode-solutions.net/products_dragon.php
	[121]	ID_68	Heterogeneous	Based on scaffolds and structural features	Leadscope Predictive Data Miner (LPDM), Leadscope, Inc., (2016): http://www.leadscope.com/
		ID_69	Heterogeneous	The top ten most common structural features linked to active compounds: benzene, 1,3-dihydroxy-; Scaffold 288; benzene, 1-alkyl-,4-amino(NH2)-; benzene, 1,2-dihydroxy-; Scaffold 297; alcohol, alkenyl-; Scaffold 576; benzene, 1-alkoxy-,4-hydroxy-; Scaffold 306; Scaffold 574. The top ten most commons structural features linked to inactive compounds: Scaffold 110; Scaffold 342; Scaffold 210; Scaffold 253; Scaffold 303; Scaffold 108; benzene, 1-alkyl-,4-halo-; halide, benzyl-; Scaffold 454; Scaffold 194	Leadscope Predictive Data Miner (LPDM), Leadscope, Inc., (2016): http://www.leadscope.com/
TBG	[52]	ID_1	PBBs	Molecular Weight (MW); Critical temperature (CT); Critical pressure (CP); Topological diameter (TD)	PaDEL [180]; Gaussian (Gaussian 09 (Gaussian Inc., Wallingford, CT, USA); ChemDraw 12.0
		ID_2	PBBs and OH-PBBs	Quadrupole moment Q_yy_ (Q_yy_); Most negative Mulliken charge number (q_−_); Frequency (Freq); TD	PaDEL [180]; Gaussian (Gaussian 09 (Gaussian Inc., Wallingford, CT, USA); ChemDraw 12.0
		ID_3	PBBs and 2OH-PBBs	q^−^; CP; TD; Topological Shape (TS)	PaDEL [180]; Gaussian (Gaussian 09 (Gaussian Inc., Wallingford, CT, USA); ChemDraw 12.0
		ID_4	PBBs, OH-PBBs, and 2OH-PBBs	q^−^; CP; TD; CT	PaDEL [180]; Gaussian (Gaussian 09 (Gaussian Inc., Wallingford, CT, USA); ChemDraw 12.0
NIS	[51]	ID_25	Heterogeneous	Calculation of 119 RDKit chemical descriptors	RDKit: Open-source cheminformatics. http://www.rdkit.org
	[104]	ID_30	Heterogeneous	Calculation of extended fingerprints with a KNIME implementation of the CDK toolkit	CDK toolkit: https://cdk.github.io/
	[119]	ID_59	Heterogeneous	Calculation of count-based Morgan fingerprints with a radius of 2 bonds and a length of 2048 bits, and of all 119 one-dimensional and two-dimensional RDKit chemical descriptors	RDKit: Open-source cheminformatics. http://www.rdkit.org
Albumin	[93]	ID_12	PFAS	PDI; GATS8v; MATS8m; QED	AlvaDesc 2.0.16 [183]
		ID_13	PFAS	Eig12_AEA(bo); DECC; X4A	AlvaDesc 2.0.16 [183]
		ID_14	PFAS	QED; PDI; GATS8v; MATS8m	AlvaDesc 2.0.16 [183]
DIO1	[51]	ID_22	Heterogeneous	Calculation of 119 RDKit chemical descriptors	RDKit: Open-source cheminformatics. http://www.rdkit.org
	[119]	ID_56	Heterogeneous	Calculation of count-based Morgan fingerprints with a radius of 2 bonds and a length of 2048 bits, and of all 119 one-dimensional and two-dimensional RDKit chemical descriptors	RDKit: Open-source cheminformatics. http://www.rdkit.org
DIO2	[51]	ID_23	Heterogeneous	Calculation of 119 RDKit chemical descriptors	RDKit: Open-source cheminformatics. http://www.rdkit.org
	[119]	ID_57	Heterogeneous	Calculation of count-based Morgan fingerprints with a radius of 2 bonds and a length of 2048 bits, and of all 119 one-dimensional and two-dimensional RDKit chemical descriptors	RDKit: Open-source cheminformatics. http://www.rdkit.org
DIO3	[51]	ID_24	Heterogeneous	Calculation of 119 RDKit chemical descriptors	RDKit: Open-source cheminformatics. http://www.rdkit.org
	[119]	ID_58	Heterogeneous	Calculation of count-based Morgan fingerprints with a radius of 2 bonds and a length of 2048 bits, and of all 119 one-dimensional and two-dimensional RDKit chemical descriptors	RDKit: Open-source cheminformatics. http://www.rdkit.org
TRHR	[51]	ID_21	Heterogeneous	Calculation of 119 RDKit chemical descriptors	RDKit: Open-source cheminformatics. http://www.rdkit.org
	[119]	ID_55	Heterogeneous	Calculation of count-based Morgan fingerprints with a radius of 2 bonds and a length of 2048 bits, and of all 119 one-dimensional and two-dimensional RDKit chemical descriptors	RDKit: Open-source cheminformatics. http://www.rdkit.org

**Table 3:** Summary and main characteristics of relevant QSARs published from January to July 2025. C: classification-based; R: regression-based; Primary: data generated as part of the same study; Secondary: data collected from the existing literature.

Model ID	Reference	Year	MIE	Algorithm	C or R	Chemical Class	Data Source Type	Data Source Literature Reference(s)
ID_2025_1	[186]	2025	Albumin	MLR	R	Phenoxyacetic acid-derived congeners	Primary	[186]
ID_2025_2	[187]	2025	TTR	RF	C	Heterogenous	Secondary	[188]
ID_2025_3	[189]	2025	TTR	LDA	C	PFAS	Secondary	[190]
ID_2025_4	[189]	2025	TTR	MLR	R	PFAS	Secondary	[190]
ID_2025_5	[191]	2025	TTR	DTC	C	PFAS	Primary	[191]
ID_2025_6	[191]	2025	TTR	MLR	R	PFAS	Primary	[191]

**Table 4:** Summary of relevant QSARs published from January to July 2025 and selected molecular descriptors, grouped by MIE.

MIE	Ref.	Model ID	Chemical class	Descriptors	Software
TTR	[187]	ID_2025_2	Heterogenous	Thirty-one descriptors sorted by permutation importance: CrippenLogP; ATSC3c; ATSC5c; C1SP3; ETA_BetaP_s; naAromAtom; ZMIC1; ATSC4m; ZMIC5; ATSC4c; hmin; hmax; ATSC2m; ATSC5m; ETA_Beta_ns_d; ATSC0m; VE1_DzZ; C1SP2; ZMIC2; ATSC1m; nHBAcc; ZMIC3; ATSC3m; ATSC2c; ETA_dAlpha_A; ETA_Shape_Y; ATSC0c; maxdssC; ZMIC4; nHBDon; ATSC1c	PaDEL descriptors from OPERA software v2.9 [192]
	[189]	ID_2025_3	PFAS	GATS3e; ATSC6p; GATS8m; MIC2	PaDEL [180]
	[189]	ID_2025_4	PFAS	piPC5; GGI9; AATSC0e	PaDEL [180]
	[191]	ID_2025_5	PFAS	SM4_D; GATS3m	AlvaDesc [182]
	[191]	ID_2025_6	PFAS	AMW; GATS7p; B10[F-F]	AlvaDesc [182]
Albumin	[186]	ID_2025_1	Phenoxyacetic acid-derived congeners	logkBMC; α; sum of HBD and HBA	ACD/Percepta software, version 1994–2012 (ACD/Labs, Advanced Chemistry Development, Inc., Toronto, ON, Canada)

## Data Availability

No new data were created or analysed in this study. The data supporting the findings of this study are included within the paper and Supplementary Materials. Further inquiries can be directed to the corresponding authors.

## Supplementary Materials

The following supporting information can be downloaded at: https://www.mdpi.com/article/10.3390/toxics13090799/s1.

## Abbreviations

The following abbreviations are used in this manuscript:

AD	Applicability domain
AdaB	Adaptive Boosting
AhR	Aryl hydrocarbon receptor
AOP	Adverse outcome pathway
ASNNs	Associative neural networks
BFR	Brominated flame retardant
BMC	Biopartitioning micellar chromatography
CAR	Constitutive androstane receptor
CART	Classification and regression trees
CP	Conformal prediction
DBP	Phenolic disinfection byproduct
DIO	Iodothyronine deiodinase
DIO1	Type 1 deiodinase
DIO2	Type 2 deiodinase
DIO3	Type 3 deiodinase
DTC	Decision Tree Classifier
DUOX	Dual oxidase
EDC	Endocrine-disrupting chemical
EU	European Union
EURL ECVAM	European Union Reference Laboratory for Alternatives to Animal Testing
FAIR	Findable, Accessible, Interoperable, Reusable
FP	Fingerprint
HPA	Hypothalamic–pituitary–adrenal
HPG	Hypothalamic–pituitary–gonadal
HPT	Hypothalamic–pituitary–thyroid
HTS	High-throughput screening
IYD	Iodotyrosine deiodinase
kNN	k-nearest neighbours
LDA	Linear discriminant analysis
LMO	Leave more out
LOO	Leave one out
LR	Logistic regression
MCT	Monocarboxylate transporter
MCT10	Monocarboxylate transporter 10
MCT8	Monocarboxylate transporter 8
MDR1	Multidrug resistance protein 1
MIE	Molecular initiating event
MLR	Multiple linear regression
MRM	Multiple regression model
MRP2	Multidrug resistance-associated protein 2
MW	Molecular weight
NAMs	New approach methodologies
NIS	Sodium iodide symporter
NN	Neural network
OATP	Organic anion transporter polypeptide
OATP1A4	Organic anion transporter polypeptide 1A4
OATP1C1	Organic anion transporter polypeptide 1C1
OECD	Organisation for Economic Co-operation and Development
PBB	Polybrominated biphenyl
PBDE	Polybrominated diphenyl ether
PCA	Principal component analysis
PCB	Polychlorinated biphenyl
PCN	Polychlorinated naphthalene
PFAS	Per- and polyfluoroalkyl substances
PFC	Poly- and perfluorinated compound
PLR	Partial logistic regression
PLS	Partial least squares
PLS-DA	Partial least squares discriminant analysis
PPAR	Peroxisome proliferator-activated receptor
PS	Prediction entropy
PXR	Pregnane X receptor
QSAR	Quantitative structure–activity relationship
RF	Random forest
SHAP	Shapley additive explanation
SMOTEENN	Synthetic minority over-sampling technique-edited nearest neighbours
SVM	Support vector machine
T3	Triiodothyronine
T4	Thyroxine
TBG	Thyroid-binding globulin
TH	Thyroid hormone
THSDC	Thyroid hormone system-disrupting chemical
TPO	Thyroperoxidase
TR	Thyroid hormone receptor
TRHR	Thyrotropin-releasing hormone receptor
TSHR	Thyroid-stimulating hormone receptor
TTR	Transthyretin
XGB	Extreme gradient boosting
